# FSP1 is a predictive biomarker of osteosarcoma cells’ susceptibility to ferroptotic cell death and a potential therapeutic target

**DOI:** 10.1038/s41420-024-01854-2

**Published:** 2024-02-17

**Authors:** Elzbieta Panczyszyn, Valentina Saverio, Romina Monzani, Mara Gagliardi, Jelena Petrovic, Jasmina Stojkovska, Licio Collavin, Marco Corazzari

**Affiliations:** 1 Department of Health Sciences and Center for Translational Research on Autoimmune and Allergic Disease (CAAD), University of Piemonte Orientale, Novara, Italy; 2 Interdisciplinary Research Center of Autoimmune Diseases (IRCAD), University of Piemonte Orientale, Novara, Italy; 3 University of Belgrade, Faculty of Technology and Metallurgy, Belgrade, Serbia; 4 Innovation Center of the Faculty of Technology and Metallurgy, Belgrade, Serbia; 5 Department of Life Sciences, University of Trieste, Trieste, Italy

**Keywords:** Bone cancer, Tumour biomarkers

## Abstract

Human osteosarcoma (OS) is a relatively rare malignancy preferentially affecting long body bones which prognosis is often poor also due to the lack of effective therapies. Clinical management of this cancer basically relies on surgical removal of primary tumor coupled with radio/chemotherapy. Unfortunately, most osteosarcoma cells are resistant to conventional therapy, with the undergoing epithelial-mesenchymal transition (EMT) giving rise to gene expression reprogramming, thus increasing cancer cell invasiveness and metastatic potential. Alternative clinical approaches are thus urgently needed. In this context, the recently described ferroptotic cell death represents an attractive new strategy to efficiently kill cancer cells, since most chemoresistant and mesenchymal-shaped tumors display high susceptibility to pro-ferroptotic compounds. However, cancer cells have also evolved anti-ferroptotic strategies, which somehow sustain their survival upon ferroptosis induction. Indeed, here we show that osteosarcoma cell lines display heterogeneous sensitivity to ferroptosis execution, correlating with the mesenchymal phenotype, which is consistently affected by the expression of the well-known anti-ferroptotic factor ferroptosis suppressor protein 1 (FSP1). Interestingly, inhibiting the activity or expression of FSP1 restores cancer cell sensitivity to ferroptosis. Moreover, we also found that: i) AKRs might also contribute to resistance; ii) NRF2 enhances FSP1 expression upon ferroptosis induction; while iii) p53 contributes to the regulation of FSP1 basal expression in OS cells.

In conclusion, FSP1 expression can potentially be used as a valuable predictive marker of OS sensitivity to ferroptosis and as a new potential therapeutic target.

## Introduction

Osteosarcoma (OS) is one of the most common malignant tumors of the skeletal system, accounting for approximately 56% of all bone cancers. OS originates from osteoid and/or immature bone produced by malignant mesenchymal cells, and thus typically affects children, adolescents, and young adults between the ages of 14-18 [1–3]. OS most commonly occurs in the metaphysis of long bones, with the highest incidence in the distal femur, proximal tibia, and proximal humerus, suggesting a link between rapid bone growth and the risk of tumor formation [4, 5]. Owing to its high malignancy rate, early metastasis, easy recurrence, and resistance to therapy, it is characterized by a high mortality rate and poor prognosis [6]. Currently, the primary treatment for OS consists of extensive surgical resection combined with neoadjuvant or adjuvant multidrug chemotherapy, with radiotherapy and immunotherapy playing increasingly important roles in comprehensive OS treatment [5, 7]. Nevertheless, the 5-year overall survival outcome of patients with localized OS remains at approximately 70%, with limited therapeutic progress over the past few decades [8, 9]. Drug resistance in OS cells is a critical factor contributing to therapeutic failure and tumor recurrence [10]. Novel classes of immunotherapy drugs, called immune checkpoint inhibitors (ICIs), have received ever-increasing interest owing to their high efficacy and reduced side effects in cancer therapy [11, 12]. In contrast to conventional cytotoxic therapies, the delivery of inhibitory signals to T cells using ICIs reprograms the adaptive immune response to promote the recognition and elimination of tumor cells. ICIs treatment has demonstrated remarkable therapeutic effects in a wide range of malignancies, such as melanoma, lung cancer, head and neck and breast cancer [12, 13]. These developments have sparked research on the potential use of ICIs in bone sarcoma treatment. However, to date, clinical trials of immune checkpoints blockade in OS have failed to elicit significant antitumor efficacy [14, 15]. The lack of improvement in the survival rates of patients with OS clearly indicates an urgent need for a better understanding of the biology of the disease onset, and for exploring novel therapeutic targets to develop new therapeutic strategies for OS treatment [4, 8].

Accumulating evidence has shown that an antitumor strategy based on the induction of non-apoptotic cell death is a promising avenue of research to overcome the existing problem of drug-resistant cancers [16]. Ferroptosis is a recently discovered form of programmed cell death that differs from apoptosis, necrosis, and autophagy by diverse sets of morphological characteristics, inducing agents, and regulatory mechanisms. It is characterized by the iron-dependent accumulation of lipid peroxides and intracellular reactive oxygen species (ROS) to lethal levels. The molecular mechanism of ferroptosis is identified by the inhibition of the cystine/glutamate antiporter (X_*C*_-) system by erastine or glutathione peroxidase 4 (GPX4) by RSL3, resulting in the intracellular accumulation of phospholipid hydroperoxides (PLOOHs or lipid-ROS) [17]. The latter molecules are considered the main executors of this cell death process and, although the precise molecular mechanism is still debated, the main hypothesis is based on their ability to destabilize the plasma membrane structure [17, 18].

Therefore, targeted regulation of ferroptosis in tumor cells might represent a fascinating and promising approach for cancer therapy [17]. Indeed, several evidence confirmed that ferroptosis plays a crucial role in limiting cancer cell growth, enhancing cell killing, and overcoming drug resistance, in many different types of cancers, such as hepatocellular carcinoma, breast carcinoma, melanoma, ovarian carcinoma, and kidney carcinoma [18–21]. However, susceptibility to ferroptosis is highly heterogeneous in different tumor cells, thus requiring a further deep understanding of the molecular mechanisms regulating the whole process.

The ferroptosis suppressor protein 1 (FSP1) dependent CoQ10 reduction system (NADH-FSP1-CoQ10) has recently been described as a GPX4-independent ferroptosis resistance circuit [19, 22]. Indeed, FSP1 reduces ubiquinone (CoQ) to ubiquinol (CoQH2), a radical-trapping antioxidant able to reduce lipid peroxides, thus inhibiting ferroptosis execution [23]. Interestingly, emerging data show that FSP1 inhibition selectively sensitizes cancer cells, characterized by enhanced expression of this factor, to ferroptosis, thus highlighting its promising prognostic role and potential therapeutic target [23–25].

In this study, we show that the heterogeneous cellular response and sensitivity of osteosarcoma cells to ferroptosis execution is directly related to FSP1 basal expression. Indeed, inhibiting the expression or activity of FSP1 efficiently re-sensitizes resistant OS cells to ferroptosis. We also provide insights into the molecular mechanism(s) regulating FSP1 basal expression in OS cells, thus indicating FSP1 as a new valuable predictive biomarker of OS sensitivity to ferroptosis and a new potential therapeutic target.

## Results

### Heterogeneous sensitivity of osteosarcoma cells to ferroptotic cell death execution

**Fig. 1:**
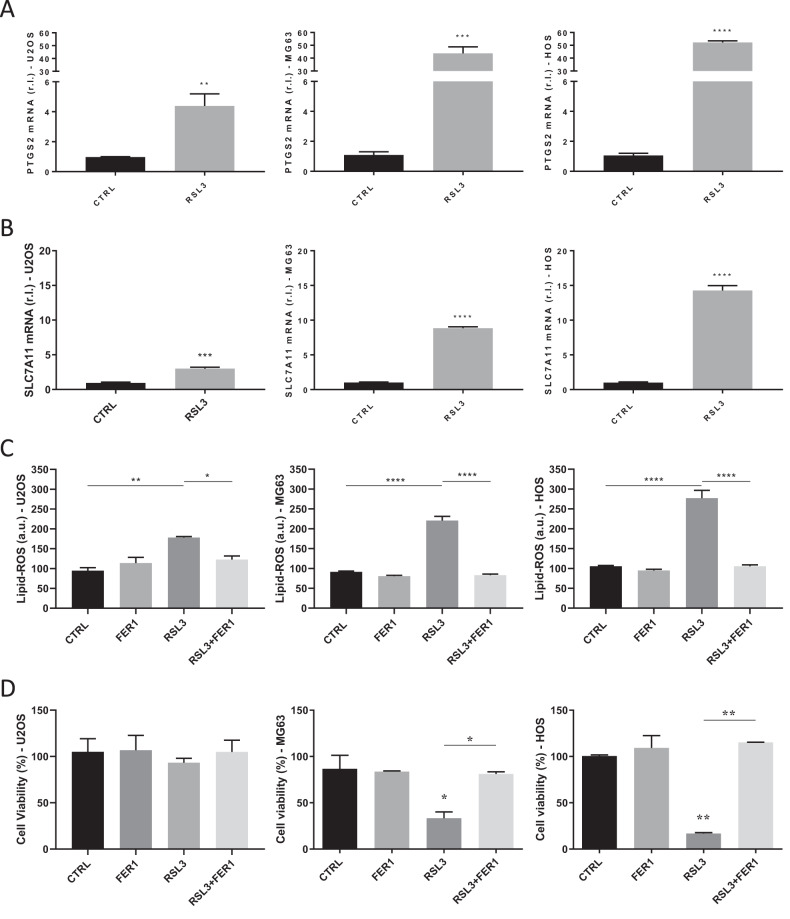
Sensitivity of osteosarcoma cells to ferroptosis induction and execution. The expression level of ferroptotic markers PTGS2 (**A**) and SLC7A11 (**B**) was evaluated in U2OS, MG63 and HOS cell lines treated or untreated for 8 h with RLS3 (0.5 μM), by qPCR. **C** The three osteosarcoma cell lines were exposed 2 h to 0.5 μM RSL3 in absence or presence of Ferrostatin-1 (FER1; 10μM) and Lipid-ROS accumulation was evaluated in cells stained with BODIPY C11, by flow cytometry. Histograms represent mean ± s.d.; *n* = 3; *p < 0.05*;* **p < 0.*01;* ****p < 0.0001. **D** The indicated osteosarcoma cell lines were exposed 18 h to RLS3 (0.5 μM) alone or in combination with Ferrostatin-1 (FER1; 10 μM) and cell viability was evaluated in AlamarBlue-stained cells. Histograms represent mean ± s.d.; *n* = 3; ns = not significant; **p < 0.01; ***p < 0.001; ****p < 0.0001.

Cancer cells are heterogeneously sensitive to ferroptotic cell death, depending on tumor type, microenvironment, and acquired mutations, rendering each tumor a unique entity, increasing the difficulties in identifying the right therapeutic regimen. Increasing our knowledge of tumor diversity will help in designing the appropriate treatment. Therefore, to evaluate the sensitivity of human osteosarcoma to ferroptosis, we evaluated the induction and execution of this nonapoptotic cell death process in a panel of three human osteosarcoma (OS) cell lines: U2OS, MG63, and HOS, using the GPX4 specific inhibitor RSL3 as pro-ferroptotic inducer. To this aim, the three cell lines were exposed to 0.5 µM RSL3 and the expression of the well-known ferroptotic markers PTGS2 and SLC7A11 [26] was evaluated after 8 h, by qPCR. The obtained results indicate a significant upregulation of both PTGS2 and SLC7A11 in cells treated with 0.5 µM RSL3, compared to untreated controls (Fig. 1A-B). Interestingly, we noted that the expression of both markers was marked in HOS, slightly lower in MG63, and significantly lower in U2OS. Next, we evaluated the production/accumulation of Lipid-ROS, the main executioners of ferroptosis. To this aim, the three cell lines were exposed (RSL3) or unexposed (CTRL) to RSL3 (0.5 µM) and Lipid-ROS were evaluated in cells stained with BODIPY C11 (2 h), by flow cytometry. As shown in Fig. 1C and in line with PTGS2 and SLC7A11 expression, we observed a huge accumulation of these molecules in HOS, slightly less in MG63 and significantly lower in U2OS, upon RSL3 exposure. Moreover, Lipid-ROS production was completely prevented in all OS cells in the presence of the specific ferroptotic inhibitor and Lipid-ROS scavenger Ferrostatin-1 (FER1; 10 µM; Fig. 1C). Then, OS cells were exposed to 0.5 µM RSL3 and cell viability was evaluated at 18 h, in cells stained with AlamarBlue. Interestingly, significant differences were observed in the execution of ferroptotic cell death among the three cell lines (Fig. 1D). Indeed, the viability of HOS cells dramatically decreased upon RSL3 exposure, to 17%, MG63 were also sensitive to treatment, with cell viability dropping to 34%, while U2OS cells did not respond to the treatment (93% of cell viability). These differences in ferroptosis execution of the three OS cell lines were better evidenced when data were plotted together (Suppl. S1). Of note, in line with Lipid-ROS production, RSL3-induced cells death was completely abrogated in cells concomitantly exposed to FER1 (compare RSL3 + FER1 vs RSL3; Fig. 1D), indicating that RSL3 specifically stimulates a ferroptotic cell death modality in OS cells, which execution requires the production/accumulation of Lipid-ROS.

Collectively, these results clearly indicate that U2OS is the most resistant, while HOS is the most sensitive to ferroptosis, in our panel of OS cell lines.

### EMT and ferroptotic sensitivity of osteosarcoma

Epithelial-to-mesenchymal transition (EMT) is an essential mechanism for the progression of osteosarcoma to detach from its original site and to gain invasive and metastatic phenotype [27]. Moreover, some evidence suggests that cancer cells that are resistant to chemotherapy or undergoing EMT may display an increased vulnerability to ferroptotic cell death [28].

**Fig. 2:**
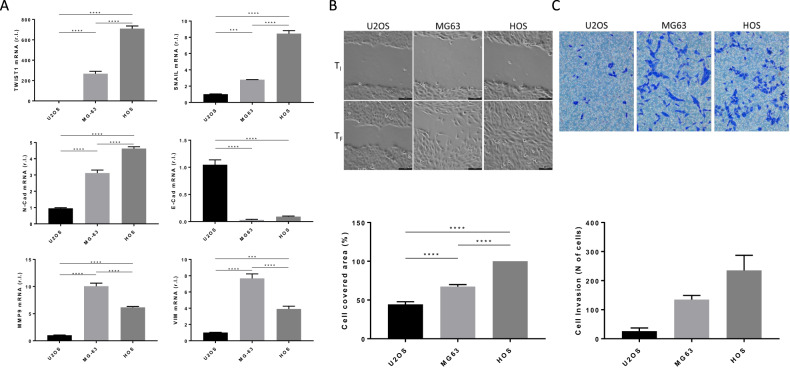
EMT and ferroptotic sensitivity of OS. **A** The basal level of indicated EMT-related genes (TWIST1, N-Cad, E-Cad, MMP9 and VIM) was evaluated in osteosarcoma cell lines by qPCR. **B** Wound-healing assays was performed by using U2OS, MG63 or HOS cells, in a time-lapse experiment (24 h, Δt = 30 min). Representative images of t_0_ and t_24_ h are reported in the upper panels, while cell-covered area (%) was reported in the bottom panel (scale bar = 10 µm). **C** Cell invasion assay was performed by using U2OS, MG63 or HOS cells, and cells were stained with 0.1% Crystal Violet (upper panels) at 48 h. Quantitative analysis of migrated cells was reported in the bottom panel. Histograms represent mean ± s.d.; *n* = 3; ***p < 0.001; ****p < 0.0001.

In order to verify if mesenchymal profile is correlated to the ferroptotic sensitivity of osteosarcoma cells, we evaluated the expression of well-known EMT markers in our panel of osteosarcoma cell lines (U2OS, MG63 and HOS), by qPCR. Specifically, we analyzed the expression of transcription factors TWIST1 and SNAIL, cell adhesion molecules N-Cadherin (N-Cad) and E-cadherin (E-Cad), matrix metalloproteinase 9 (MMP9), and intermediate filament protein Vimentin (VIM). The expression of all EMT markers, except for E-Cad, was significantly enhanced in the ferroptotic sensitive HOS cell line, while decreased in mild sensitive MG63, and lower in the most resistant U2OS. (Fig. 2A). As expected, the epithelial marker E-Cad was instead overexpressed in the most resistant U2OS, while it was expressed to a lesser extent in both MG63 and HOS. Therefore, to verify that the most sensitive HOS, showing the most pronounced expression of mesenchymal markers, are effectively the most metastatic, we evaluated both migration and invasiveness of U2OS, MG63, and HOS. To this aim, cells were grown until confluence, scratch wounds were generated through a pipet tip, and cell migration was recorded in a time lapse experiment within 24 h (initial time, Ti and final time, Tf). Quantification of the wounded area revealed that ferroptotic sensible HOS cells migrated faster compared to both MG63 and the ferroptotic resistant U2OS. Therefore, after 24 h of the scratch, the cell-free area was completely covered by HOS cells (Fig. 2B and Supplementary MOV1-6).

Finally, cell invasion ability was also evaluated through the Transwell Migration Assays. Results reported in Fig. 2C clearly indicate that HOS cells show the highest invasiveness compared to both MG63 and U2OS, with the latter being the less invasive. Overall, these data clearly indicate a correlation between EMT and ferroptosis resistance of osteosarcoma cells.

### Involvement of AKRs in osteosarcoma resistance to ferroptosis

**Fig. 3:**
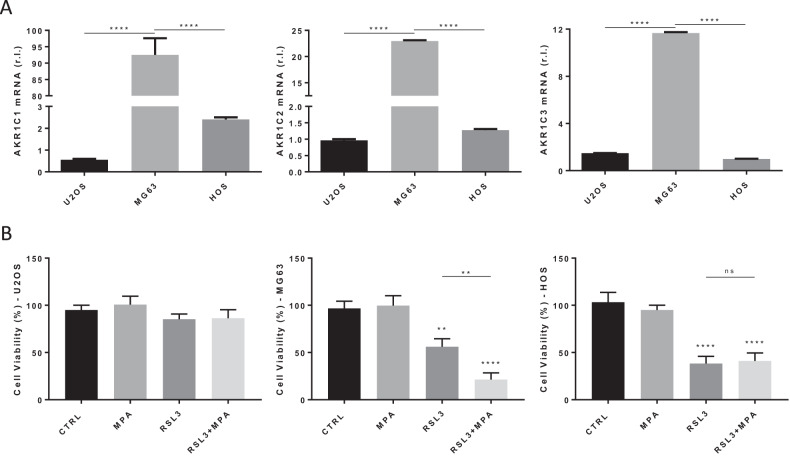
Ferroptotic sensitivity of osteosarcoma cells and AKRs. **A** The expression level of AKR1C1, AKR1C2 and AKR1C3 was evaluated in U2OS, MG63 and HOS cell lines, by qPCR. **B** The indicated osteosarcoma cell lines were untreated (CTRL) or treated with MPA (10 μM) or RLS3 (0.5 μM) alone or in combination (RLS3 + MPA), and cell viability was evaluated at 18 h in AlamarBlue-stained cells. Histograms represent the mean ± s.d.; *n* = 3; ns = not significant, **p < 0.01; ****p < 0.0001.

To identify the potential molecular mechanism(s) responsible for the heterogeneous sensitivity of OS cells to ferroptosis execution, we evaluated the involvement of known factors previously described to be able to confer cancer cell resistance to ferroptosis. Indeed, we recently identified three members of the aldo-keto reductase family of enzymes (AKR1C1–3), responsible for metastatic melanoma resistance to ferroptosis execution [20]. Therefore, we evaluated the expression of the three ARKs in our panel of OS cells, by qPCR. Data reported in Fig. 3A clearly indicate a concomitant enhanced basal expression of AKR1C1, AKR1C2 and AKR1C3 in MG63 compared to both U2OS and HOS cells, which does not fit with ferroptosis sensitivity shown in Supplementary S1. Next, to better define the potential role of AKRs activity in the response of OS cells to pro-ferroptotic treatment, cells were exposed to RLS3 alone or in combination with the AKRs inhibitor MPA [20, 21]. Results reported in Fig. 3B indicate no significant changes in both U2OS and HOS cell sensitivity to RSL3, when AKRs activity was inhibited (compare RSL3 + MPA and RSL3 columns of left and right panels of Fig. 3A-B). On the contrary, MPA enhanced the sensitivity of MG63 cells to RSL3 (compare RSL3 and RSL3 + MPA columns of Fig. 3B, middle panel). Collectively, these data indicate that OS cells are characterized by the heterogeneous basal expression of AKRs, which play a minor role in their resistance to ferroptosis execution.

Next, we evaluated the potential involvement of the GCH1/BH4 axis, able to mitigate the ferroptotic process [29]. Analysis of GCH1 expression in the three OS cell lines revealed a heterogeneous basal and stimulated (0.5 µM RSL3, 8 h) expression of this factor (Suppl. S2). In particular, GCH1 basal expression was similar in the most resistant U2OS and most sensitive HOS, while stimulated expression was not affected in U2OS while increased (5 folds) in HOS cells. On the contrary, GCH1 basal expression was very low in MG63, while reaching about the basal level of both U2OS and HOS, upon RSL3 stimulation. To further investigate the role of GCH1 in OS resistance to ferroptosis, data from patients with sarcoma were analyzed by the GEPIA2 web platform, and the level of GCH1 mRNA was analyzed, revealing no significant change in tumors compared to normal tissues (Suppl. S3). Collectively, these data indicate that the GCH1/BH4 anti-ferroptotic system seems not involved in the regulation of ferroptotic sensitivity/resistance of osteosarcoma cells.

### FSP1 plays a key role in OS resistance to ferroptosis induction

**Fig. 4:**
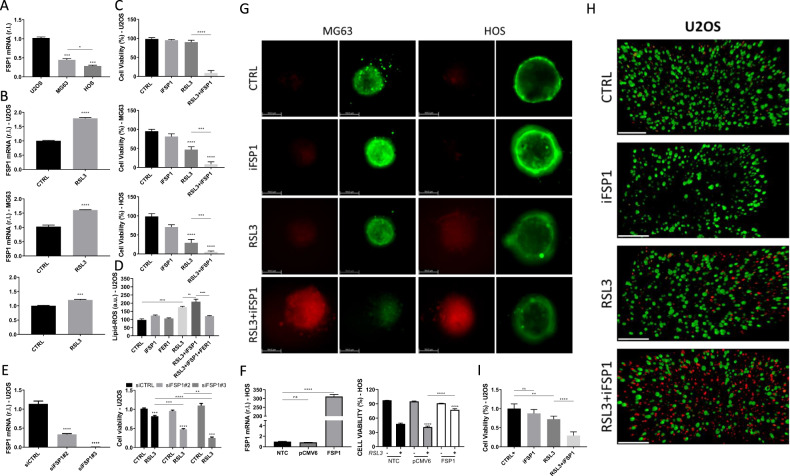
FSP1 confers resistance to ferroptosis execution in osteosarcoma cells. **A** The expression level of FSP1 was evaluated in U2OS, MG63 and HOS cell lines, by qPCR. **B** U2OS, MG63 and HOS cell lines were exposed 8 h to RLS3 (0.5 μM) and the expression of FSP1 was evaluated by qPCR. **C** The indicated osteosarcoma cell lines were untreated (CTRL) or treated with iFSP1 (6 μM) or RLS3 (0.5 μM) alone or in combination (RLS3 + iFSP1), and cell viability was evaluated at 18 h, in AlamarBlue-stained cells. **D** Lipid-ROS accumulation was evaluated in U2OS cells exposed as indicated (4 h), by FACS analysis of BODIPY C11 stained cells. 6μM iFSP1; 0.5μM RSL3; 10μM FER1. Histograms represent mean ± s.d. Experiments were performed at least three times. ****p < 0.0001; **p < 0.01. **E** U2OS cells were transiently transfected with siRNAs targeting FSP1 (siFSP1#2 and siFSP1#3), while a scrambled sequence was used as a control (siCTRL). FSP1 expression was evaluated 48 h post-transfection by qPCR (left panel); cells were exposed to RLS3 (0.5 μM) for 18 h and cell viability was quantified in AlamarBlue-stained cells (right panel). Histograms represent mean ± s.d.; *n* = 3; *p < 0.05; **p < 0.01; ***p < 0.001; ****p < 0.0001. **F** HOS cells were untransfected (NTC) or transiently transfected with an empty vector (pCMV6) or a vector coding for FSP1. The expression of FSP1 was evaluated after 48 h, by qPCR (left panel). Next, cells were untreated or treated with RSL3 (0.5μM), and cell viability was evaluated after 18 h in AlamarBlue stained cells. Histograms represent the mean ± s.d.; *n* = 3; ns not statistically significant; **p < 0.01; ****p < 0.0001. **G** Live/Dead assay was performed on spheroids generated by MG63 (left panels) or HOS (right panels), by using Calcein-AM (green) and Propidium Iodide (PI, Red), untreated (CTRL) or treated with iFSP1 (6 μM) or RLS3 (0.5 μM) alone or in combination (RLS3 + iFSP1). Scale bar = 164.5 μm. Representative images of *n* = 3 independent experiments. **H** Live/Dead assay was performed on alginate microfibers with immobilized U2OS cells by using Calcein-AM (green) and Propidium Iodide (PI, Red), on untreated (CTRL) or treated with iFSP1 (6 μM) or RLS3 (0.5 μM) alone or in combination (RLS3 + iFSP1) (scale bar = 200 μm). Representative images of *n* = 3 independent experiments. (**I**) Alginate microfibers with immobilized U2OS cells were treated as in **E**, and cell viability was evaluated after 18 h, in AlamarBlue-stained cells. Histograms represent mean ± s.d.; *n* = 3; ns non statistically significant; **p < 0.01; ****p < 0.0001.

Ferroptosis suppressor protein 1 (FSP1), also known as apoptosis-inducing factor mitochondrial 2 (AIFM2), or AMID, is a recently described inhibitor of ferroptosis execution, working in parallel but independent of GPX4 [10, 29]. Therefore, to investigate the potential role of FSP1 in the resistance of OS cells to ferroptosis, we evaluated its basal levels in our panel of cells. Data reported in Fig. 4A show that U2OS cells are characterized by the highest, while HOS show the lowest level of FSP1 mRNA, with MG63 cells characterized by intermediate levels. This expression pattern was confirmed by western blotting analysis of FSP1 protein levels in the three OS cell lines (Suppl. S4). Interestingly, combining the expression of PTGS2 and SLC7A11 with cell viability of RSL3-treated cells, and basal expression of FSP1 of each OS cell line, we observed an interesting correlation between PTGS2 and SLC7A11 stimulated expression, which is inversely correlated to both basal FSP1 and sensitivity to ferroptosis (Suppl. S5). These results raised the hypothesis that basal expression of FSP1 might be used as a potential predictor biomarker of OS sensitivity to ferroptosis execution.

The potential involvement of FSP1 in the resistance of OS cells to ferroptosis execution is further supported by data showing an RLS3-stimulated up-regulation of FSP1 that is particularly evident in the most resistant U2OS, while less evident in MG63, and milder in the most sensitive HOS (Fig. 4B).

To test our hypothesis, we inhibited the activity of FSP1 by means of the specific inhibitor iFSP1 (6 µM) and exposed all OS cell lines to 0.5 µM RSL3, for 24 h. Cell viability, evaluated in AlamarBlue-stained cells, revealed an enhanced susceptibility to ferroptosis execution of all OS cell lines (Fig. 4C). Importantly, FSP1 inhibition completely reverted the phenotype of resistant U2OS cells, rendering these cells highly sensitive to ferroptosis execution (Fig. 4C upper panel). The key anti-ferroptotic role played by FSP1 in U2OS cells was also highlighted by measuring the accumulation of Lipid-ROS in cells exposed to RSL3 in presence of iFSP1 (Fig. 4D). Indeed, the combination enhanced the accumulation of lipid peroxides (compare RSL3 with RSL3 + iFSP1), which was completely abrogated in the presence of ferrostatin-1 (RSL3 + iFSP1 + FER1; Fig. 4D). To confirm these results, we inhibited the expression of FSP1. Indeed, U2OS were transiently transfected with siRNA oligonucleotides targeting FSP1 (siFSP1#2 and siFSP1#3), while a scrambled sequence (siCTRL) was used as a control, and FSP1 expression was then evaluated by qPCR, after 24 h (Fig. 4E, left panel). Cells were then exposed to 0.5 µM RSL3, and cell viability was evaluated after further 24 h. Results shown in Fig. 4E (right panel) indicate a significant reduction in the viability of FSP1-silenced U2OS cells when exposed to RSL3, the extent of which correlates perfectly with the FSP1 levels. Finally, we ectopically expressed FSP1 in the most sensitive HOS, characterized by low expression of this protein (compared to both MG63 and U2OS; Fig. 4F, left panel), and evaluated its sensitivity to RSL3-induced ferroptosis. As shown in Fig. 4F (right panel), HOS overexpressing FSP1 are significantly resistant to ferroptosis execution, compared to both control (pCMV6) and parental (NTC) cells.

In addition, we evaluated whether AKRs and FSP1 play a cooperative anti-ferroptotic role, at least in OS cells, in which both factors are significantly expressed. To this end, we exposed MG63 cells to RSL3 alone and in combination with both MPA and iFSP1, and cell viability was evaluated after 24 h. Data reported in Supplementary S6 show that the concomitant inhibition of AKR and FSP1 consistently enhances the sensitivity of MG63 to ferroptosis. Ferrostatin-1 (FER-1) was used as a specific ferroptotic inhibitor.

Although representing simple and low-cost models, 2D cell culture systems might not represent the in vivo tumor conditions, since proper 3D tumor structure, cell-cell, and cell-extracellular matrix interactions are known to be crucial for cancer cell proliferation, differentiation, as well as responsiveness to stimuli and drug metabolism. Indeed, recent reports from anti-cancer drug research have highlighted the advantage of 3D models over standard 2D cell cultures, showing significant differences in terms of drug efficacy [30–33]. Therefore, we evaluated the susceptibility of OS cells to RSL3-induced ferroptosis in 3D OS spheroids. To this aim, spheroids were generated from MG63 or HOS cells, by means of low attachment cell culture plates, and ferroptosis was induced by RSL3 alone or in combination with iFSP1. Cell viability was then evaluated after 4 h of treatment by using the Live/Dead assay based on Calcein-AM (green) and PI (red) double staining. Consistently with results from 2D cell cultures, images acquired by a THUNDER 3D Cell Imager microscope revealed that HOS cells are more sensitive than MG63, and that FSP1 inhibition consistently increases the susceptibility of both cell types to RSL3 treatment (Fig. 4G). U2OS cells were not included in this study, due to their inability to form spheroids. To overcome this inconvenience, U2OS cells were immobilized in alginate hydrogels. Therefore, U2OS cells were immobilized in alginate-based 3D microfibers and unexposed or exposed to RSL3 or iFSP1 alone or in combination, as reported for spheroids. Cell viability was evaluated by both Live/Dead assay or AlamarBlue. Simple extrusion of the cell/alginate suspension (4×10^6^ cells/mL, 2.8% w/w alginate) directly into the gelling bath resulted in the formation of uniform alginate microfibers (diameter 745 ± 90 µm) with immobilized U2OS cells. Data reported in Fig. 4H and I clearly show that FSP1 inhibition consistently increases the susceptibility of also the most resistant U2OS cells to RSL3-induced ferroptosis in the 3D culture model.

### NRF2 marginally regulates FSP1 expression in osteosarcoma cells

Due to the key role played by FSP1 in the ferroptosis resistance of osteosarcoma cells and its potential use as a predictor marker, we analyzed the molecular mechanism(s) regulating its basal expression. A recent report identified FSP1 as a transcriptional target of NRF2, the key antioxidant master gene and factor mediating ferroptosis and radiation resistance in lung cancer [32, 34]. Importantly, NRF2 is known to be frequently deregulated in human OS tumors, thus it would not be surprising if it was also the case of OS cells included in this study [34].

**Fig. 5:**
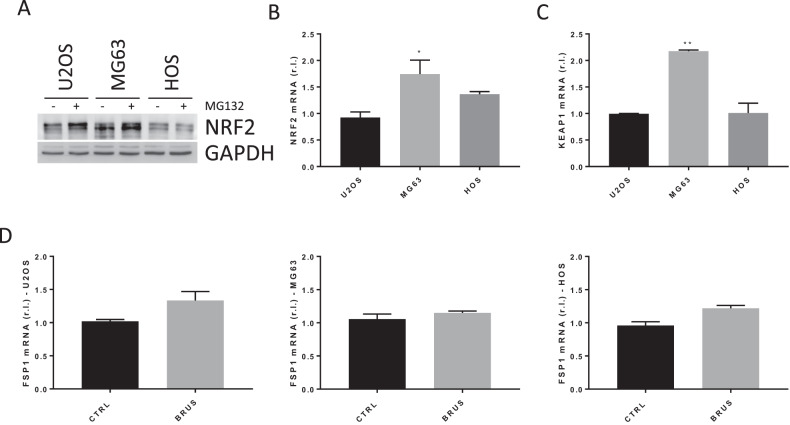
NRF2 marginally regulates FSP1 expression upon ferroptosis induction in ferroptotic resistant osteosarcoma cells. **A** The NRF2 basal protein expression was evaluated in osteosarcoma cells by western blotting analysis. GAPDH was used as a loading control. The expression of NRF2 was also evaluated in the same OS cells, by qPCR analysis (**B**), together with the basal expression of KEAP1 (**C**). **D** The indicated osteosarcoma cells were exposed 8 h to Brusatol (BRUS, 50 nM) and the expression of FSP1 was evaluated by qPCR. Histograms represent mean ± s.d.; *n* = 3; *p < 0.05; **p < 0.01; ****p < 0.0001.

To test whether NRF2 is involved in the basal expression regulation of FSP1, we evaluated both the protein and mRNA levels of this transcription factor in our panel of osteosarcoma cell lines. NRF2 expression in U2OS is very similar to that observed in HOS, while it is slightly higher in MG63, at both protein level and mRNA (Fig. 5A-B, respectively). Similar results were obtained when evaluating the expression of Keap1, a well-known negative regulator of NRF2 (Fig. 5C). These results do not fit with FSP1 expression, thus potentially indicating that NRF2 is not involved in the regulation of basal FSP1 expression, at least in OS cells tested in this study. To confirm these results, we inhibited the activity of NRF2 by using Brusatol (BRUS, 50 nM) and evaluated the expression of FSP1 in U2OS, MG63 and HOS cells, compared to untreated controls, by qPCR. Indeed, data reported in Fig. 5D show that NRF2 inhibition did not reduce basal FSP1 levels in any of the cell lines.

However, the role of NRF2 in the ferroptotic process has been previously described, with this factor involved in the regulation of both general antioxidant target genes, due to oxidative stress imposed by ferroptosis induction, and ferroptotic specific factors [35]. Therefore, to verify the integrity of NRF2 signaling pathways and its prompt activation upon ferroptosis stimulation, indicating the presence of wt NRF2 in our OS cells, we evaluated the expression of the oxidative stress-related HO-1 and NQO1, and ferroptosis-related GPX4 and SLC7A11, in cells unexposed or exposed to RSL3 or BRUS alone and in combination. Data reported in Supplementary S7 show the NRF2-dependent up-regulation of these genes upon RSL3 exposure, which was abrogated by BRUS.

Collectively, these data indicate that NRF2 is activated early during ferroptosis induction, but does not contribute to FSP1 basal expression.

In an attempt to understand the potential mechanisms regulating FSP1 basal expression in ferroptosis-resistant osteosarcoma, we analyzed the putative promoter region of FSP1, by the free web platform PROMO-ALGGEN, searching for potential TFs able to regulate FSP1 expression. Interestingly, among other, we identified two potential consensus regions (responsive elements; RE) for the TFs E2F1 and p53, respectively (data not shown). We focused on these two TFs since p53 was previously suggested to potentially regulate FSP1 expression, and known to be involved in ferroptosis regulation, while E2F1, frequently deregulated in human osteosarcoma, has still an ambiguous role in ferroptosis regulation [36, 37].

To test this hypothesis, we evaluated the expression of E2F1 in our OS cell lines, by qPCR. Data reported in Supplementary S9A show a heterogeneous expression of E2F1, which was higher in U2OS (used as control, 1.13 ± 0.12), while decreasing in MG63 (0.81 ± 0.02) and HOS (0.39 ± 0.02). Interestingly, the E2F1 expression pattern seems to correlate with that of FSP1, in the same cells (compare Suppl. S9A with Fig. 3A), thereby supporting our hypothesis. Next, we knocked-down the expression of E2F1 in the most resistant U2OS cells, characterized by enhanced expression of both E2F1 and FSP1, by using a specific siRNA (siE2F1), while a scrambled siRNA sequence was used as a control (siCTRL). However, as shown in Suppl. S9B-C, E2F1 knockdown had no impact on FSP1 expression. Therefore, these findings indicate that E2F1 is not essential for FSP1 expression in osteosarcoma cells.

### p53 contributes to regulate FSP1 basal expression in osteosarcoma cells

**Fig. 6:**
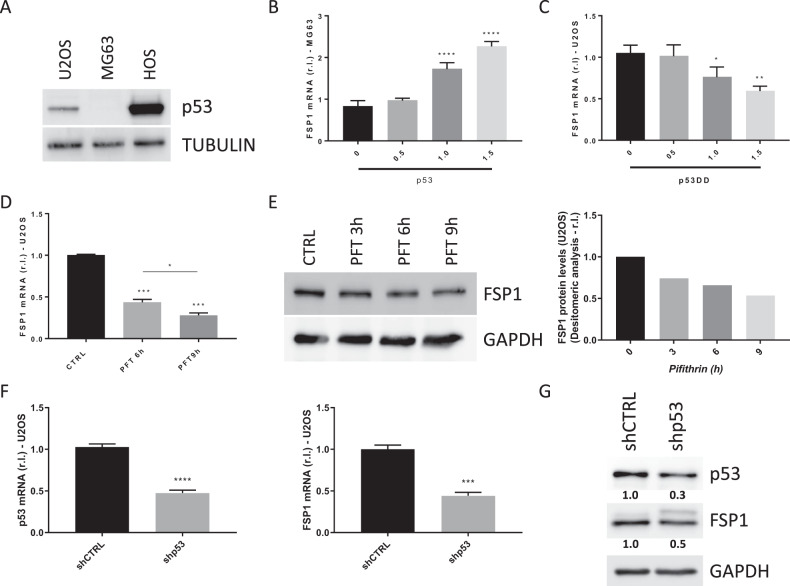
p53 regulates FSP1 basal expression. **A** Basal level of p53 was evaluated in osteosarcoma cell lines by western blotting analysis. TUBULIN was used as a loading control. **B** MG63 cells were transiently transfected with increasing amounts of a vector coding for human p53 (0, 0.5, 1.0 and 1.5 μg), while (**C**) U2OS cells were transiently transfected with increasing amounts of a vector coding for human p53DD (0, 0.5, 1.0 and 1.5 μg), and the expression of FSP1 was evaluated by qPCR. U2OS cells were exposed to Pifithrin (PFT 10 μM) at for 3, 6 or 9 h, and the expression of FSP1 was evaluated by qPCR (**D**) or western blotting (**E**) analysis. GAPDH was used as loading control. Densitometric analysis was performed and reported in the right panel. U2OS cells were infected with lentiviral particles carrying a specific shRNA targeting p53, while a scrambled sequence was used as control (shCTRL). The expression of p53 and FSP1 was evaluated 48 h post-infection by qPCR (**F**) or western blotting (**G**) analysis. GAPDH was used as loading control. Histograms represent mean ± s.d.; *n* = 3; *p < *0.05;* **p < 0.01; ***p < 0.001; ****p < 0.0001. A densitometric analysis of IB reported in panel G was performed using the Image Lab software (bio-Rad), using GAPDH as a loading control, and data were reported under each corresponding band.

As reported above, FSP1 promoter analysis also revealed the presence of a putative p53-responsive element. U2OS cells are characterized by a wild-type p53 [38], MG63 cells are p53-null [39], while HOS cells are characterized by a mutant p53R156P with an inactivated transactivation domain (TA) [40]. Indeed, western blotting analysis of p53 expression in the three OS cell lines revealed a low expression in U2OS (typical of functional p53), no expression in MG63 and a consistent expression in HOS (frequently observed with non-functional p53) (Fig. 6A). Next, we transiently overexpressed wild type p53 [41] in the p53-null MG63 cells, and evaluated the levels of FSP1. As evidenced in Fig. 6B, MG63 cells exhibited a wt p53 dose-dependent increase of FSP1 gene expression. Then, we impaired the activity of wt p53, by transiently expressing an increasing amount of a p53DD (dominant negative) [42] in p53 wt U2OS cells. Data reported in Fig. 6C show p53DD dose-dependent downregulation of FSP1 mRNA levels. To confirm these results, we inhibited the activity of wt p53 by exposing U2OS cells to Pifithrin (PFT, 10 μM), a known inhibitor of p53 transcriptional activity [43]. Results shown in Fig. 6D-E clearly show a time-dependent downregulation of FSP1 at both mRNA and protein levels, in cells exposed to PFT.

Finally, to further confirm these results, we inhibited the expression of wt p53 in U2OS by infecting cells with lentiviral particles carrying a specific shRNA targeting p53 (shp53), while a scrambled shRNA sequence was used as a control (shCTRL). Our results indicate that inhibiting the expression of wt p53 (Fig. 6F, left panel) decrease the basal expression of FSP1, at both mRNA (Fig. 6F, right panel) and protein (Fig. 6G) levels. Collectively, these results indicate that p53 contributes to regulate the basal expression of FSP1 in osteosarcoma cells.

## Discussion

Cancer is the second leading cause of death worldwide, accounting for nearly 10 million deaths by 2020 [44], and in contrast to others, cancer death rates have decreased by 27% in the past 20 years (2001-2020). This important progress reflects consistent advances in treatment; however, some patients affected by specific cancer types do not take advantage of these improvements, remaining, indeed, relatively incurable [45, 46], such as osteosarcoma. It is a relatively rare malignancy of mesenchymal origin that accounts for the majority of bone cancers. Despite significant advances in surgery and combined therapies for OS over the past few years, the overall survival rate of patients affected by this malignancy has not increased significantly [7]. Thus, extensive research aimed at better understanding the molecular mechanisms, therapeutic targets, and potential drugs for targeted OS therapy is urgently required. Here, we explored a potential strategy to kill osteosarcoma cells based on a recently described new form of programmed cell death, known as ferroptosis. It is characterized by iron-dependent accumulation of lipid peroxides, the executioners of this form of non-apoptotic cell death, providing new treatment opportunities for cancers resistant to conventional therapies [10, 47].

Interestingly, we successfully induced early stages of ferroptosis in a panel of three human osteosarcoma cell lines, as confirmed by the prompt and significant upregulation of specific markers, such as PTGS2 and SLC7A11, in response to exposure to RSL3, while the execution of the process was highly heterogeneous, indicating the activation of signaling pathways conferring resistance. Of note, the rate of induction of the above-mentioned ferroptotic markers reflected the sensitivity to treatment. Moreover, the production and accumulation of Lipid-ROS also reflected the sensitivity of OS cell lines to ferroptosis, with Ferrostatin-1 completely preventing both lipid peroxide production and cell death.

Cancer cells have evolved a heterogeneous repertoire of molecular pathways aimed at sustaining cancer cell proliferation and resistance to pro-death stimuli, thus inhibiting apoptosis, necroptosis, and also ferroptosis [48].

Among these, recently, members of the AKRs family of enzymes (specifically AKR1C1-3), have been described to reduce the intracellular and ferroptosis-related accumulation of lipid peroxides, thus inhibiting ferroptosis execution, and conferring resistance to human metastatic melanoma cells [20]. However, the expression of AKRs did not fit the observed profile of OS sensitivity to ferroptosis, although inhibiting their activity increased ferroptosis sensitivity of the cell line characterized by enhanced basal expression of the three enzymes. These data indicate that AKRs might contribute to ferroptosis resistance of OS cells, depending on their basal expression, but do not represent the main factor conferring resistance.

The GTP cyclohydrolase 1 (GCH1) and its metabolic products, tetrahydrobiopterin (BH4) and dihydrobiopterin (BH2), have been reported to protect cancer cells from ferroptosis. GCH1 is the rate-limiting enzyme in the synthesis of BH4, which is an essential cofactor to produce aromatic amino acids, neurotransmitters, and nitric oxide [49]. BH4 is able to confer protection against ferroptosis execution by reducing oxidized phospholipids [50]. Indeed, GCH1 overexpression in mouse fibroblasts significantly inhibited ferroptosis execution by RSL3 treatment, independently of GPX4 [50]. Therefore, stimulus-dependent enhanced expression of GCH1 can be used as a biomarker for the activation of GCH1/BH4 anti-ferroptotic signaling pathways. However, no activation of this antiferroptotic pathways was observed in OS-resistant cell lines, while upregulation of GCH1 was evidenced in the most sensitive cell line, thus confirming that this mechanism is not involved in the resistance of osteosarcoma to ferroptosis execution.

Several cancer types, such as thymoma (THYM), pancreatic adenocarcinoma (PAAD), and lymphoid neoplasm diffuse large B-cell lymphoma (DLBC), are characterized by dysregulated expression of another anti-ferroptotic factor, FSP1 (Suppl. S10), which uses ubiquinone (also known as CoQ10) to reduce lipid-ROS, thus blocking ferroptosis [22, 51]. Interestingly, FSP1 basal expression, at both mRNA and protein levels, directly correlated with OS cells resistance to ferroptosis. Moreover, a further increase in FSP1 expression was evident in ferroptosis-resistant cells, upon RSL3 exposure. It should be noted that the inhibition of FSP1 activity or gene expression consistently abrogated resistance to ferroptosis execution in all tested OS cells. Similar results were obtained in OS cells cultured under 3D conditions, thus confirming that this factor confers OS resistance to ferroptosis. Therefore, we identified FSP1 as a novel potential prognostic factor and molecular target for reverting ferroptosis resistance in OS. Indeed, FSP1 overexpression conferred resistance to RSL3 in the most sensitive OS cell line.

As mentioned above, ferroptosis execution relies on the production and accumulation of ROS and, particularly, lipid-ROS, with the latter highly reactive molecules considered the main executioners of the death process. Cells react to this stress condition by up-regulating and activating the key antioxidant-related transcription factor NFR2, which, in turn, regulates the expression of different classes of target genes, such antioxidant (such as HO-1, NQO1, CAT and SOD) and ferroptosis-related genes (such as GPX4, GCLc, ACLS4, and SLC7A11) [52]. Moreover, NRF2 expression is frequently dysregulated in human osteosarcoma tissues, and its expression is also associated with poor outcomes [34, 53]. Indeed, we confirmed dysregulated basal NRF2 in OS cell lines, and upon ferroptosis induction, the expression of well-characterized anti-oxidant and ferroptotic genes were positively regulated, indicating an intact NRF2 signaling pathway.

Interestingly, among the ferroptosis-related genes regulated by NRF2 it has also recently been included FSP1 [23]. However, our data clearly indicate that NRF2 positively regulates the expression of FSP1, particularly in resistant cell lines, upon ferroptosis induction, while inhibiting the activity of this TF has no significant impact on basal FSP1 levels.

Analyzing the promoter region of the FSP1 gene, we identified, among others, two potential and interesting responsive elements targeted by E2F1 and p53. Indeed, E2F1 was found heterogeneously expressed in OS cells, with no clear correlation with basal FSP1 expression, and knocking down its expression did not affect basal FSP1 levels. Therefore, we concluded that this TF is not involved in basal FSP1 gene expression regulation, at least in OS cells.

On the other hand, the importance of p53 as genome guardian, in regulating cell cycle/death and preventing transformation is well-known [54]. Furthermore, accumulating evidence indicates that p53 is also tightly involved in the regulation of ferroptotic cell death, with emerging data indicating that its mutational status also affects the sensitivity of cancer cells to pro-ferroptotic chemotherapeutic drugs [55]. In this context, we found that p53 is actively involved in the regulation of basal FSP1 expression in osteosarcoma cells. Of note, we found that expressing the transcription factor in the p53null MG63 cell line increased basal FSP1 expression, in a dose-dependent manner, while down-regulating its expression or inhibiting the transactivation activity in the p53wt U2OS cells resulted in FSP1 down-regulation, in a dose- and time-dependent manner.

Altogether, these data indicate that both p53 and NRF2 regulate the expression of FSP1, with the former TF contributing to regulate the basal expression whereas the latter mediates FSP1 up-regulation under pro-oxidative/pro-ferroptotic conditions.

The current therapy for osteosarcoma consists of surgical resection, chemotherapy, and radiation. Despite these therapeutic options, primary bone cancer frequently causes death by pulmonary metastasis [56].

Metastatic osteosarcoma is very often further resistant to current standard therapy, worsening the already poor prognosis. Epithelial-mesenchymal transition (EMT) is reported as a critical event in cancer, transforming primary tumor cells into invasive and metastatic entities. In this regard, overexpression of EMT-marker was associated with increased invasion of osteosarcoma cells and increased mortality and morbidity of patience [57]. It is also interesting to note that recent evidence indicates that although tumors characterized by enhanced EMT, frequently induced by anti-tumor treatments, are resistant to conventional treatments, they show enhanced sensitivity to ferroptosis, compared to primary tumors, with low EMT [58].

Indeed, the expression of well-known EMT markers was significantly enhanced in the ferroptotic sensitive HOS cell line, while decreased in the mild sensitive MG63, and was lower in the most resistant U2OS. Moreover, the ferroptotic-sensitive HOS cell line showed the most pronounced invasiveness and ability to migrate compared to both MG63 and the ferroptotic-resistant U2OS.

In conclusion, the mesenchymal profile of OS cells is correlated with ferroptotic sensitivity, suggesting that EMT markers and basal FSP1 levels might be used bona fide to predict the sensitivity of this tumor to ferroptosis, thus indicating ferroptosis as a new valuable anticancer treatment for patients affected by OS.

## Materials and Methods

### Cell culture and treatments

Human osteosarcoma cell lines – U2OS, MG63 and HOS – were purchased from ‘Biological Resource Center ICLC Cell bank, Core facility IRCCS Ospedale Policlinico San Martino, Genova (IT)’, were maintained in Dulbecco’s modified Eagle’s medium (DMEM, EuroClone), supplemented with 10% fetal bovine serum (FBS, EuroClone), 2 mM L-glutamine (Merck), 1% penicillin/streptomycin (Merck), at 37 °C in a humidified incubator with 5% CO_2_. Mycoplasma testing was routinely performed each month by using the Venor®GeM Classic (Minerva-BiolAbs;Berlin, GE)

Cells were treated with 0.5 µM RSL3 (Merck), 10 µM Ferrostatin-1 (Merck), 6 µM iFSP1 (BioVision), 50 nM Brustatol (Cayman Chemicals); 10 µM MPA (Merck), or 10 µM Pifithrin (Merck), as indicated.

### Cell viability

Cell viability was measured using AlamarBlue™ reagent (Bio-Rad) according to manufacturer’s instructions. Briefly, 15×10^3^ cells/well were plated in 24-well plates, treated as indicated, cell medium was discarded and an appropriate amount of AlamarBlue reagent was added. Cells were incubated 4 h, and fluorescence was monitored (530–560 nm excitation, and 590 nm emission wavelength) using a TECAN automation platform.

### Lipid peroxides evaluation

Briefly, 1.5 × 10^5^ cells were treated as indicated and harvested at the indicated time points. Then, cells were pelleted, washed with PBS, resuspended in BODIPY C11 (2 μM in PBS; Invitrogen), incubated at 37 °C for 15 min in the dark, and 10.000 events were acquired by using a FACS Symphony cytometer (Becton-Dickinson). Data analysis was performed using the Flowing Software.

### Spheroids generation and live/dead assay

3-5×10^3^ HOS or MG63 cells were seeded in each well of a PrimeSurface®96U ultra-low attachment plate (S-BIO) and spheroid generation was monitored every day by using a THUNDER 3D Cell Imager (Leica). On day 4, spheroids were untreated (Ctrl) or treated with RSL3 or iFSP1 alone or in combination (RSL3 + iFSP1) for 4 h. Cell viability was then evaluated by Calcein-AM (0.1 µM, Dojindo) and Propidium Iodide (PI 50 ng/mL, ThermoFisher Scientific) staining. Briefly, spheroids were incubated with the Calcein/PI staining mix for 30 minutes, at 37 °C, washed in PBS and rinsed with complete medium. Images were recorded by THUNDER 3D Cell Imager.

### Production of alginate microfibers with immobilized U2OS cells and cell viability assay

A suspension of U2OS cells was mixed with 3.5% w/w sodium alginate solution to obtain a final concentration of 2.8% w/w alginate and 4×10^6^ cells/mL. The cell-alginate suspension was then manually extruded through a blunt-edge stainless steel needle (25 G, Small Parts Inc., USA) into a gelling solution containing Ca^2+^ (0.18 M) to obtain alginate hydrogel microfibers with immobilized cells. After 15 minutes of gelation, the resulting microfibers were washed with cell culture medium, transferred into T-25 flasks filled with fresh medium (1.5 g of microfibers in 15 mL of cell culture medium), and cultivated in a humidified atmosphere at 5% CO_2_ and 37 °C. The experiment lasted for 5 days, and 40% of the medium was exchanged on the 3^rd^ day.

On day 5, 1 cm of microfibers with immobilized cells were transferred into each well of a µ-Slide 8-well (Ibidi), supplemented with 400 µl of complete medium, and exposed or unexposed to iFSP1 (6 µM), or RSL3 (0.5 µM) alone or in combination (RSL3 + iFSP1), for 18 h. Then, cell viability was evaluated by incubating fibers with a Calcein-AM/PI staining mix (0.1 µM/50 ng/mL, respectively) for 30 minutes, at 37 °C. Fibers were washed in PBS and rinsed with a complete medium, and images were recorded by a Leica TCS SP8 Confocal microscope. Alternatively, a 3 cm piece of microfiber was transferred into each well of a 24-well plate supplemented with 1 mL of complete medium and treated as reported above. Cell viability was quantified using AlamarBlue, as described above.

### qPCR

Total RNA was isolated by using TripleXtractor reagent (Grisp) and ExcelRT Reverse Transcriptase (Grisp) was used to produce cDNA, by using 2 μg of total RNA. Quantitative PCR (qPCR) reactions were performed by using the Excel-Taq FAST qPCR SybrGreen (Grisp) and a CFX96 thermocycler (Bio-Rad). Primer sequences were designed by using the online IDT Pri-merQuest Tool software (IDT; https://eu.idtdna.com/Primerquest/Home/Index), and sequences reported below [59].

**Table:** 

*Name*	*Sequence*
PTGS2	GCCTGGTCTGATGATGTATG/GTATTAGCCTGCTTGTCTGG
SLC7A11	CTGGGTTTCTTGTCCCATATAA/GTTGCCCTTTCCCTCTATTC
AKR1C1	GCCGTGGAGAAGTGTAAAG/CAGACAGGCTTGTACTTGAG
AKR1C2	GGGTTCCACCATATTGATT/CACTGCCATCTGCAATCT
AKR1C3	CAGAGGTTCCGAGAAGTAAAG/CCAACCTGCTCCTCATTATT
FSP1	CCTGCCCTTCTCTCATCTTA/GTCCTCATAGGCCTGGATAG
NRF2	CCTGCCCTTCTCTCATCTTA/GTCCTCATAGGCCTGGATAG
KEAP1	GAAAGTCCACGTCTCTGTTT/CGTCCTGCACAACTGTATC
E2F1	GAAAGTCCACGTCTCTGTTT/CGTCCTGCACAACTGTATC
p53	TGTACCACCATCCACTACA/TGTTCCGTCCCAGTAGATTA
TWIST	CAGGTACATCGACTTCCTCTA/CATCCTCCAGACCGAGAA
N-Cad	GATGAAACGCCGGGATAAA/CTTCTTCTCCTCCACCTTCT
MMP9	CCATCCACGTCGTCCTTATG/CGCTGGGCTTAGATCATTC
L34	GTCCCCGAACCCTGGTAATAGA/GGCCCTGCTGACATGTTTCTT
E-Cad	CCCTTCACAGCAGAACTAAC/CACCTCTAAGGCCATCTTTG
VIM	CCAGCTAACCAACGACAAA/TCCTCTCTCTGAAGCATCTC
SNAIL	GATGAGGACAGTGGGAAAG/CCAAGGAAGAGACTGAAGTAG
HO-1	AGCTCTTCTGGGAAGTAGAC/CCTCCCTGTACCACATCTAT
GPX4	AGCTCTTCTGGGAAGTAGAC/CCTCCCTGTACCACATCTAT
NQO1	GGATGAGACACCACTGTATTT/CTCCTCATCCTGTACCTCTT
GCH1	GTGTATGGTAATGCGAGGTG/GAACTCTTCCCGAGTCTTTG

L34 mRNA level was used as an internal control, and the comparative Ct method (ΔΔCt) was used for relative quantification of gene expression [29].

### Western blotting analysis

Proteins were isolated by using a RIPA Buffer supplemented with a protease inhibitor cocktail (Merck), and an equal amount of proteins (20 µg) were subjected to an SDS-PAGE, and electroblotted onto nitrocellulose membranes (Bio-Rad). Membranes were blocked 1 h by using 5% non-fat dry milk (Merck) in PBS plus 0.1% Tween20 (Merck), and incubated with the indicated primary antibodies in blocking solution, overnight at 4 °C: anti-FSP1(1:1000, ProteinTech), anti-p53 (1:500, Santa Cruz Biotechnology), anti-NRF2 (1:1000, Cell Signaling Technology), anti-E2F1(1:500, Santa Cruz Biotechnology), anti-GAPDH (1:500, Santa Cruz Biotechnology). Detection was achieved using HRP-conjugated secondary antibodies (1:5000; Jackson ImmunoResearch) and visualized by SuperSignal West Pico Plus (ThermoFisher Scientific). Images were acquired by using a ChemiDoc Touch Imaging System (Bio-Rad) and analyzed by Image Lab software (Bio-Rad) [60].

### Small interfering RNA (siRNA)

siE2F1, siFSP1, and non-targeting scramble (siCTRL, used as negative control) siRNA oligoribonucleotides were obtained from Merck. Briefly, 25 × 10^4^ cells/well were seeded in 6-well plate and transfected with 25pmol of each siRNA, using Lipofectamine®RNAiMAX (ThermoFisher Scientific) reagent, as recommended by the supplier. Cell culture medium was replaced with fresh complete medium after 24 h, cells were lysed after further 24 h, and gene expression analysis was performed by qPCR, as described above [61].

### Plasmid transfection

An empty (pCMV6) vector or a vector coding for FSP1 (OriGene; #RC204934) was used to ectopically express FSP1 in HOS cells. To this aim, 25×10^4^ cells/well were transfected with 1 μg of DNA in 6 well plates by using JetPRIME (Polyplus) for 8 h, as recommended by the supplier. Cells were lysed after further 48 h, and gene expression was verified by qPCR.

Wild type [41] (wt) or dominant negative (DD; Addgene #25989 [42]) p53 encoding vectors were also used. A total of 25×10^4^ cells/well were transfected with 0.5, 1 or 1.5 μg of DNA in 6 well plates by using JetPRIME (Polyplus) for 8 h, as recommended by the supplier. Cells were lysed after further 48 h, and gene expression analysis was performed by qPCR, as described above [20].

### Lentiviral generation and infection

Co-transfection of lentiviral vectors (pLKO-shP53 and shCtrl, pLKO-shSCRAMBLE; 10 μg), Vesicular stomatitis virus G protein expression plasmid (psMD2; 2.5 μg), and psPAX2 plasmid (carrying gag, pol and rev genes; 7.5 μg) were performed using HEK293T packaging cell line, by a calcium phosphate protocol [47 = 61]. Supernatants with lentiviral particles were harvested 48 h later. These supernatants were used to infect U2OS. Quantitative PCR (qPCR) analysis was used to assess p53 downregulation at 48 h after infection.

### Gene expression profiling interactive analysis (GEPIA)

The analysis was performed by using the free online web platform at http://gepia.cancer-pku.cn/index.html, which details are available at http://gepia.cancer-pku.cn/help.html [62]. Briefly, the expression of GCH1 was evaluated in human sarcoma, SARC, data set matched with ‘TCGA normal and GTEx data’ set (normal tissue), with a |Log2FC| Cutoff of 1, a p-value Cutoff of 0.01, and results showed using a log2(TPM + 1) log scale [20].

### Promoter analysis

The identification of putative transcription factor binding sites (TFBS) in FSP1 promoter DNA sequence was performed by using the web platform PROMO-ALGGEN, available at: https://alggen.lsi.upc.es/cgi-bin/promo_v3/promo/promoinit.cgi?dirDB=TF_8.3. The 1355 bp upstream sequence of the known ATG site was screened.

### Wound healing assay

U2OS, MG63 and HOS cell lines were plated in 6-well plates and allowed to grow until they reached 100% confluence. The cell layer was then gently scratched through the central axis using a p200 sterile plastic tip. The width of the healing monolayer wound was recorded over 24 h in a time lapse experiment (Δt = 30 min) using a THUNDER 3D Cell Imager system (Leica).

### Invasion assays

U2OS, MG63 and HOS cell lines, at 5×10^3^ cells/well, were seeded into the upper chambers of Transwell-plates in medium without FBS. FBS (Sarsted, Nümbrecht; Germany) in the bottom chamber was used as a chemoattractant. Cells were incubated at 37 °C for 48 h, and migrated cells on the lower membrane of each insert were fixed in 70% ethanol for 10 min, and stained with Crystal Violet 0.1% for 10 min, at room temperature. Images were acquired by a THUNDER 3D Cell Imager system (Leica), and cells were counted by using ImageJ software.

### Statistical analysis

Experiments were performed in triplicate and repeated at least three times, and statistical analysis was performed using GraphPad software (GraphPad Software; GraphPad Prism 6). Student’s t test or ANOVA was used to determine statistical significance. A *p* value of equal to or less than 0.05 was considered significant. mRNA expression levels were represented as ‘fold change’, r.l. relative levels. Histograms represent mean ± SD; **** *p* < 0.0001; *** *p* < 0.001; ** *p* < 0.01; * *p* < 0.05; ns non-significant.

### Supplementary information

Author changes

Supplementary Figures

Original Data

Supplementary Information

MOV1

MOV2

MOV3

MOV4

MOV5

MOV6

MOV7

MOV8

MOV9

MOV10

## Data Availability

All data are available in the main text or the supplementary materials.
