# Supplementary material for: FSP1 is a predictive biomarker of osteosarcoma cells’ susceptibility to ferroptotic cell death and a potential therapeutic target
Source: Cell Death Discov. 2024 Feb 17;10:87. doi: 10.1038/s41420-024-01854-2 (PMC10874395; doi:10.1038/s41420-024-01854-2)
Supplement: Supplementary file 2 — Supplementary Figures [file 41420_2024_1854_MOESM2_ESM.pdf]

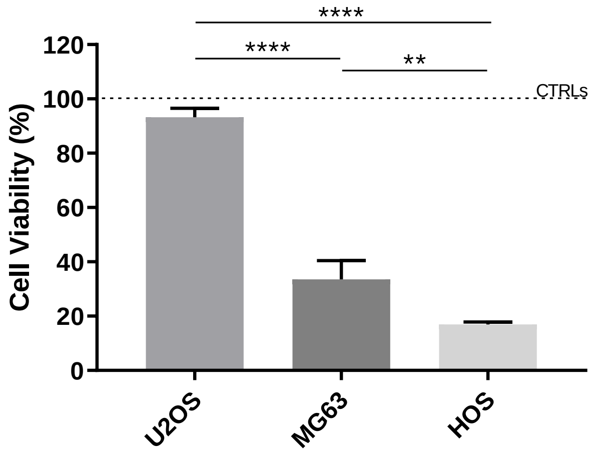

Supplementary S1. OS sensitivity to ferroptosis. The sensitivity of the indicated OS cell lines to RSL3 reported in figure 1 was compared. Histograms represent mean ± s.d. of experiments repeated three times. **** $p < 0.0001$; *** $p < 0.001$

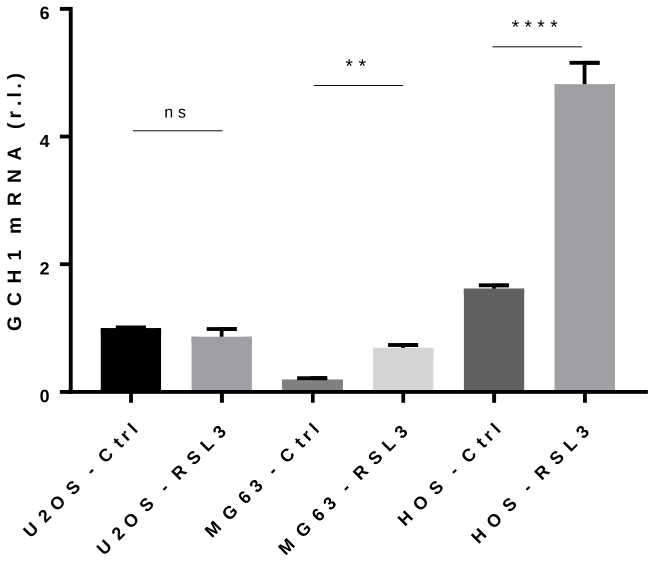

Supplementary S2. The GCH1/BH4 anti-ferroptotic axis is not involved in OS resistance to ferroptosis execution. OS cell lines were unexposed or exposed 4h to RSL3 (0.5 μM) and expression of GCH1 was evaluated by qPCR. Results are expressed as fold change and basal expression of U2OS was used as control (set to 1.0). Histograms represent mean ± s.d.; experiments were performed at least three times. **** $p < 0.0001$; ** $p < 0.01$; ns = not statistically significant.

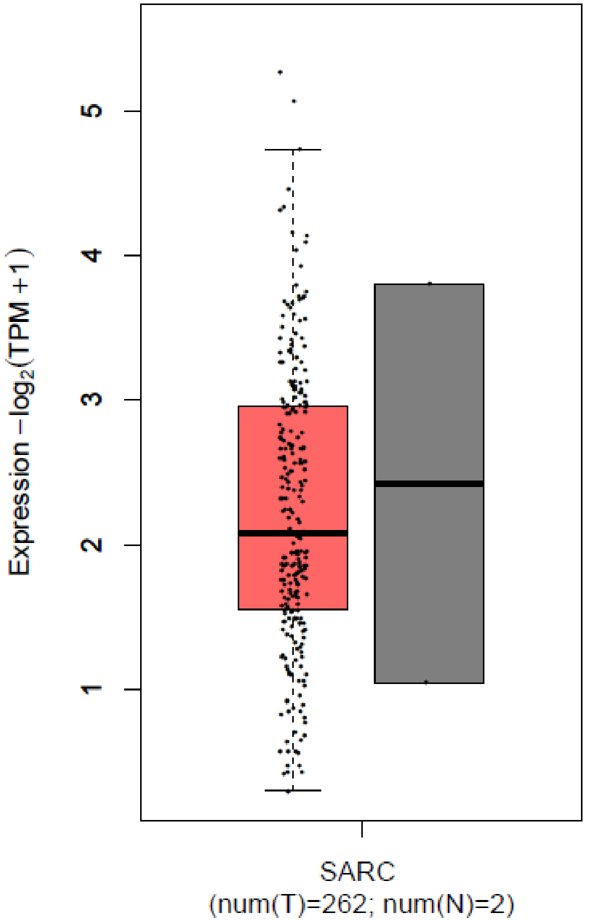

Supplementary S3. GCH1 expression. GCH1 expression of human sarcoma (SARC; Red) vs normal adjacent tissues (Gray) was evaluated by the Gene Expression Profiling Interactive Analysis tool (GEPIA) [http://gepia2.cancer-pku.cn/].

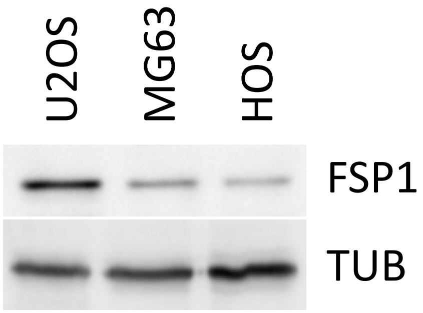

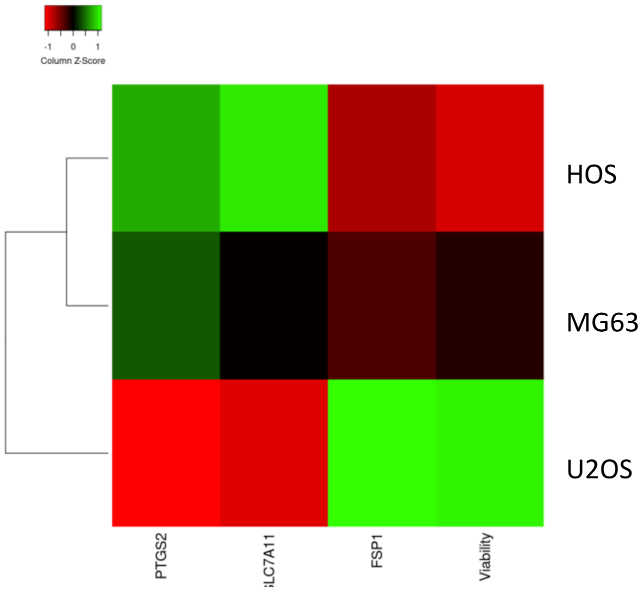

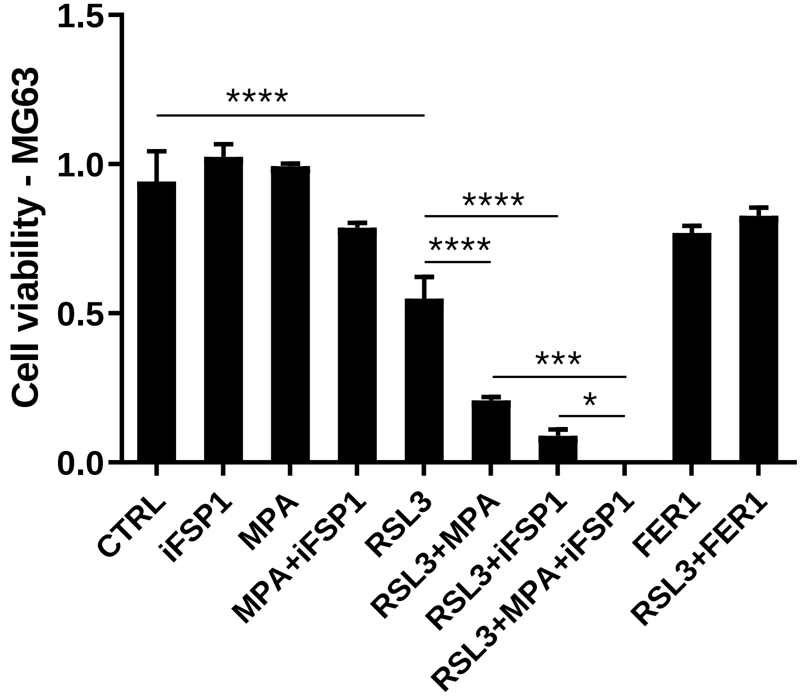

Supplementary S4. FSP1 anti-ferroptotic factor expression. Basal expression of FSP1 protein was evaluated in the indicated osteosarcoma cell lines by western blotting analysis. Tubulin was used as loading control. Representative images of three independent experiments.

Supplementary S5. Ferroptotic markers expression and OS sensitivity to ferroptosis. Ferroptosis-stimulated expression of PTGS2 and SLC7A11 was combined with FSP1 basal expression and cell sensitivity to ferroptosis of HOS, MG63 and U2OS cell lines. The resulting heatmap clearly shows a direct correlation between PTGS2 and SLC7A11 and between FSP1 and sensitivity to ferroptosis. Interestingly, PTGS2/SLC7A11 expression is inversely related to FSP1/sensitivity.

Supplementary S6. FSP1 and AKRs cooperative anti-ferroptotic role in MG63. Cell viability was evaluated in MG63 cells tretated as indicated, and reported below. 5μM iFSP1; 10μM MPA; 0.5μM RSL3; 10μM FER1. Histograms represent mean ± s.d. Experiments were performed at least three times. **** $p < 0.0001$; *** $p < 0.001$; * $p < 0.05$.

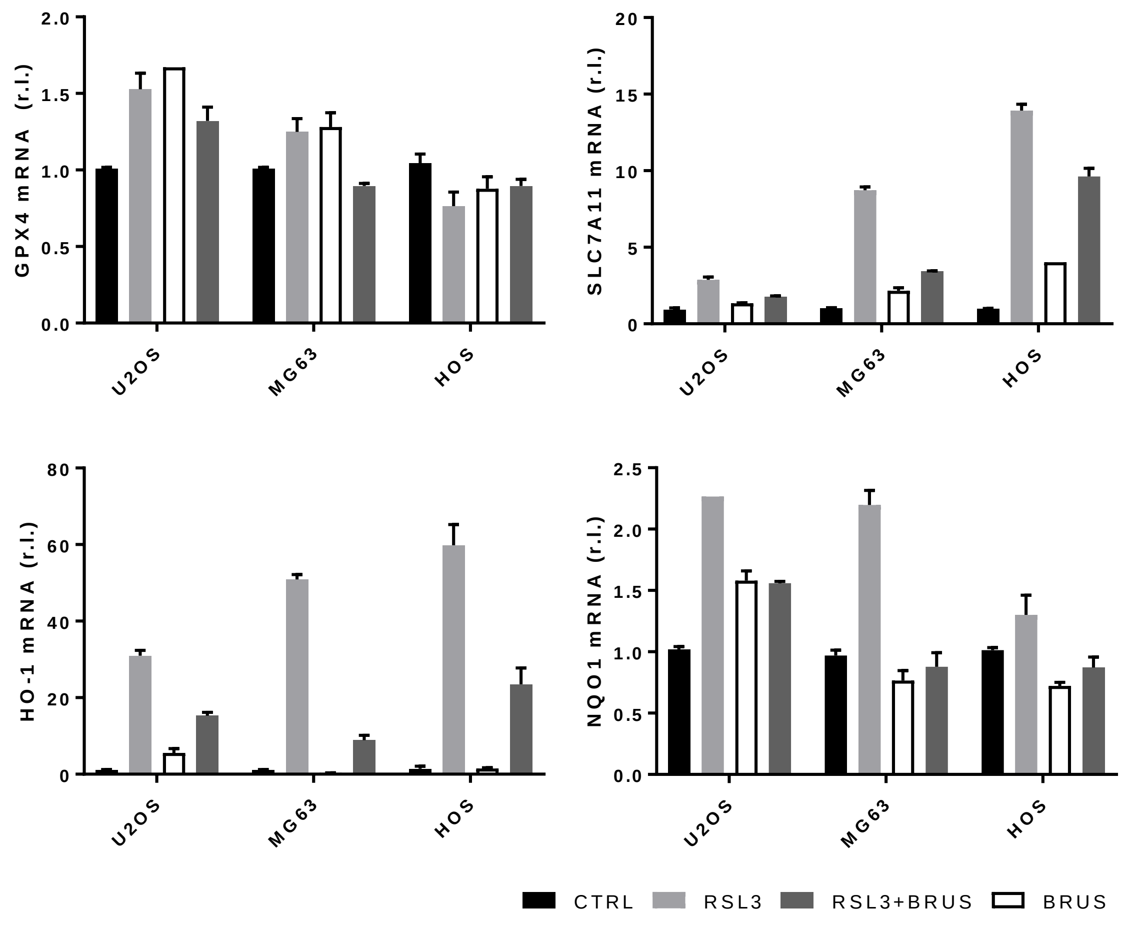

Supplementary S7. Expression of NRF2 gene targets. The expression of the indicated NRF2 targets was evaluated in the indicated osteosarcoma cell lines exposed to 50 mM Brusatol (BRU) or 0.5μM RSL3 alone or in combination (BRU+RSL3), by qPCR. Histograms represent mean ± s.d. Experiments were performed at least three times.

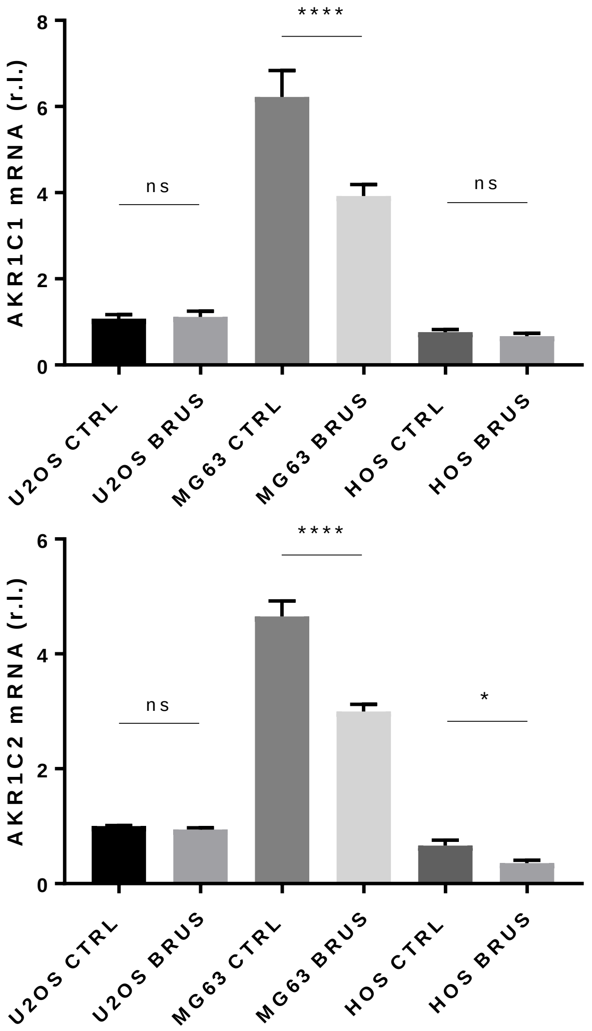

Supplementary S8. NRF2 controls AKRs expression. Cells were untreated or treated 4h with Brusatol (BRU; 50nM), and AKRs expression was evaluated by qPCR. Histograms represent mean ± s.d. Experiments were performed at least three times. Ns = not statistically significant; * p < 0.05; **** p > 0.0001.

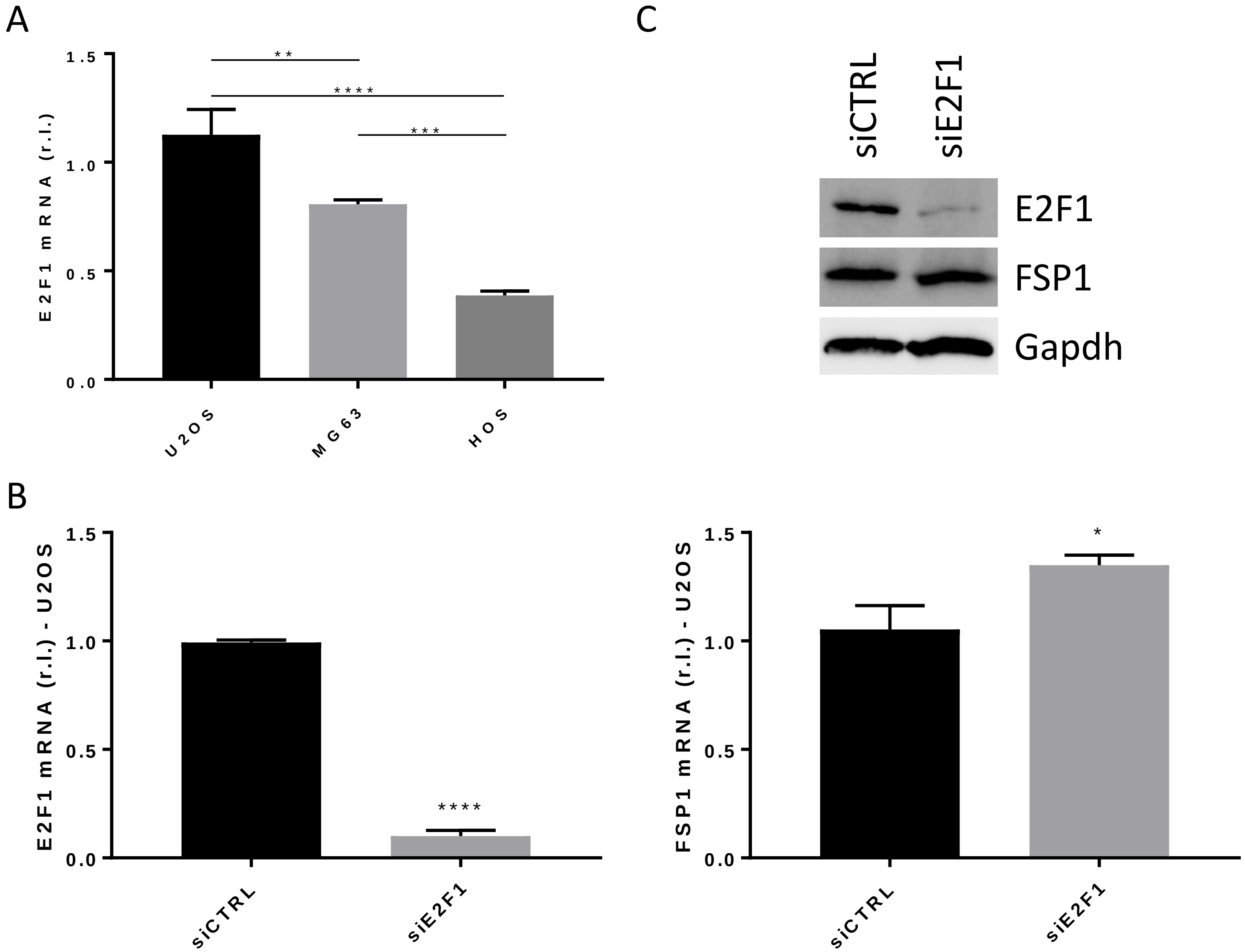

Supplementary S9. FSP1 expression is E2F1-indipendent in osteosarcoma cells. (**A**) E2F1 expression was evaluated in osteosarcoma cells, by qPCR. U2OS cells were transiently transfected with a specific siRNA targeting E2F1 (siE2F1), while a scrambled sequence was used as control (siCTRL). E2F1 and FSP1 expression was evaluated 48h post-transfection, by both qPCR (**B**) and western blotting analysis (**C**). Gapdh was used as loading control. Histograms represent mean ± s.d.; n = 3; * *p <0.05; ** p <0.01; *** p <0.001; **** p <0.0001*.

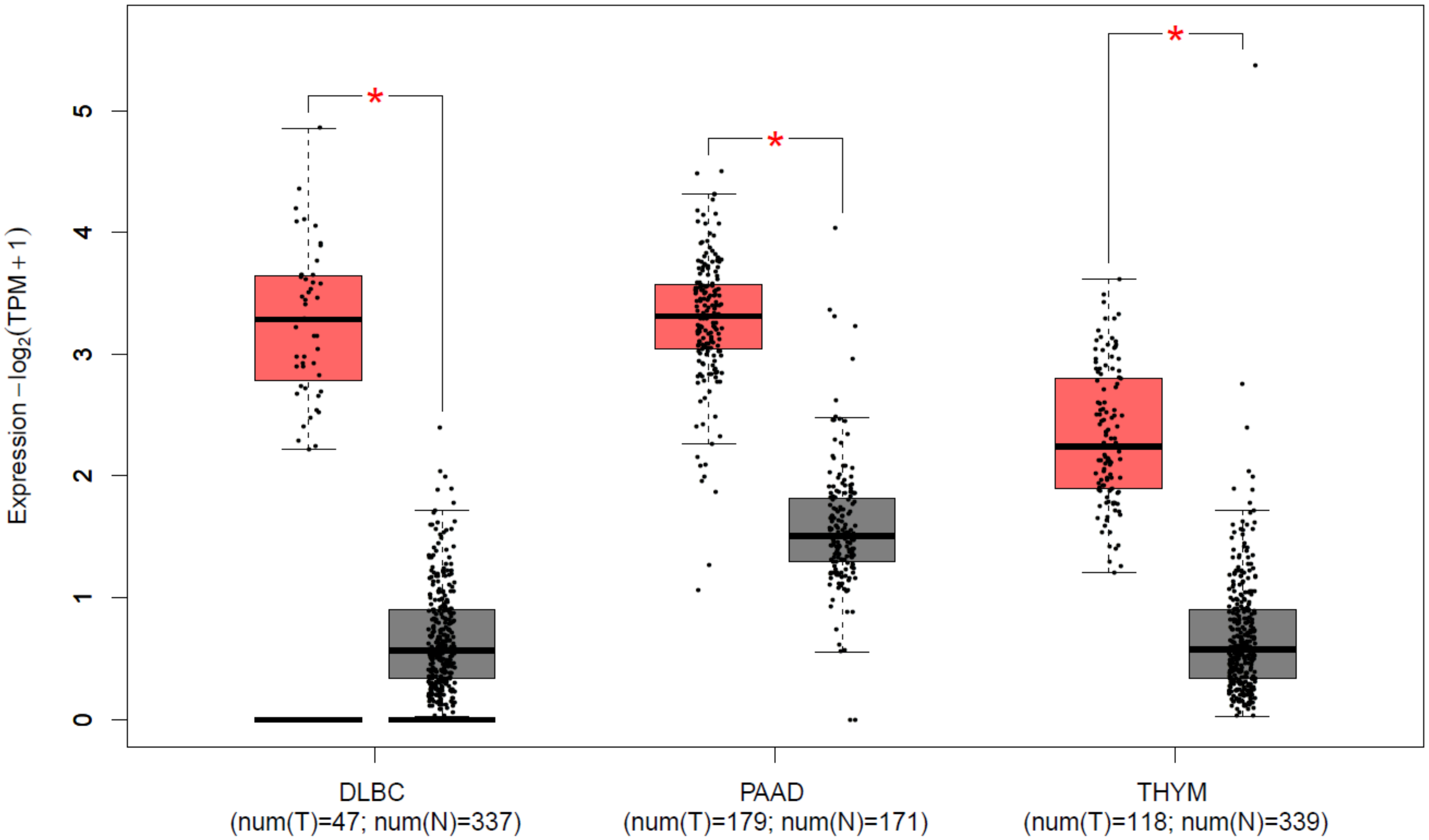

Supplementary S10. FSP1 expression. FSP1 expression of human Lymphoid Neoplasm Diffuse Large B-cell Lymphoma (DLBC), Pancreatic Adenocarcinoma (PAAD), and Thymoma (THYM) (Red) vs normal adjacent tissues (Gray) was evaluated by the Gene Expression Profiling Interactive Analysis tool (GEPIA): * $p < 0.01$ [http://gepia2.cancer-pku.cn/].
