# Supplementary material for: FSP1 is a predictive biomarker of osteosarcoma cells’ susceptibility to ferroptotic cell death and a potential therapeutic target
Source: Cell Death Discov. 2024 Feb 17;10:87. doi: 10.1038/s41420-024-01854-2 (PMC10874395; doi:10.1038/s41420-024-01854-2)
Supplement: Supplementary file 3 — Original Data [file 41420_2024_1854_MOESM3_ESM.pdf]

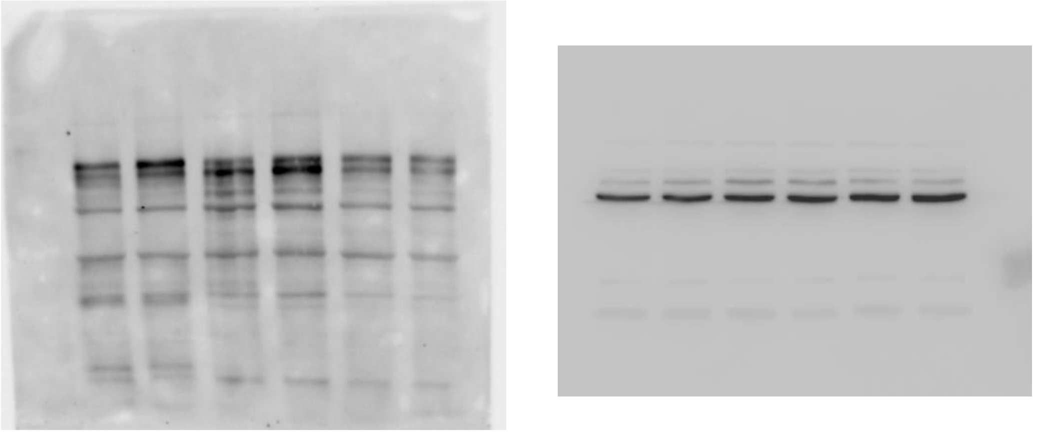

NRF2 Gapdh

Figure 5A

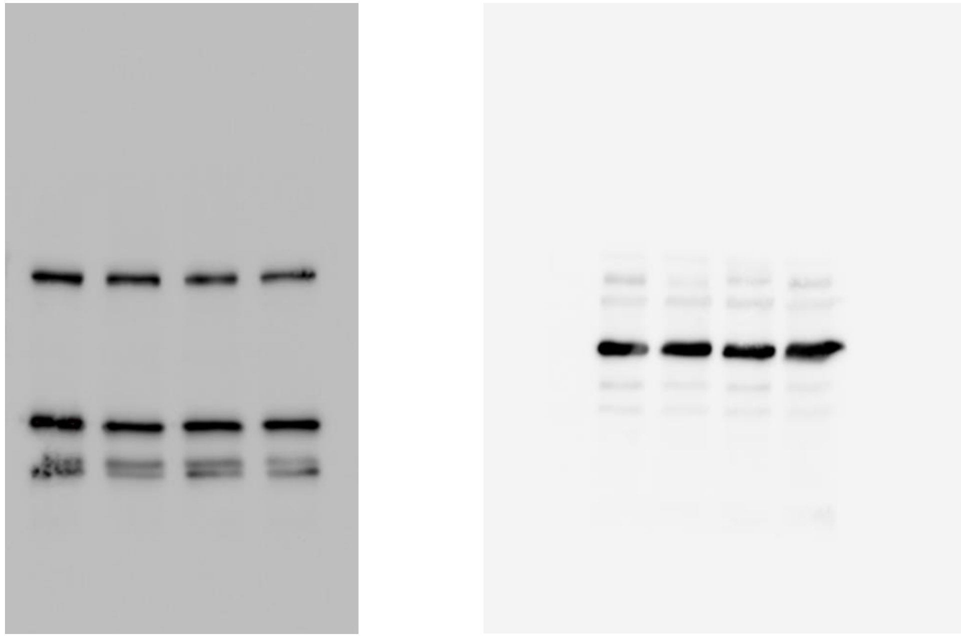

FSP1 Gapdh

Figure 6E

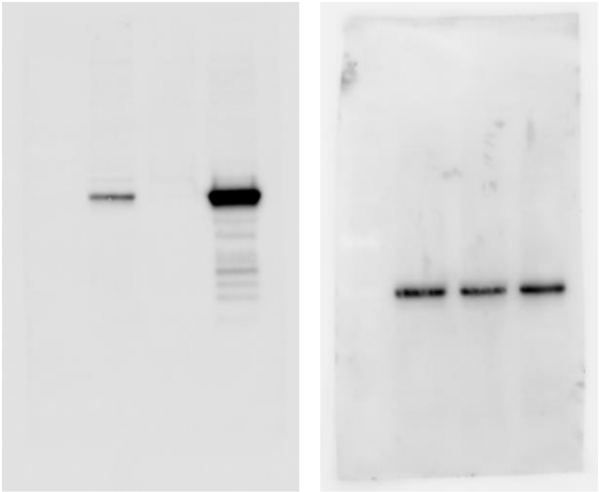

p53 Tubulin

Figure 6A

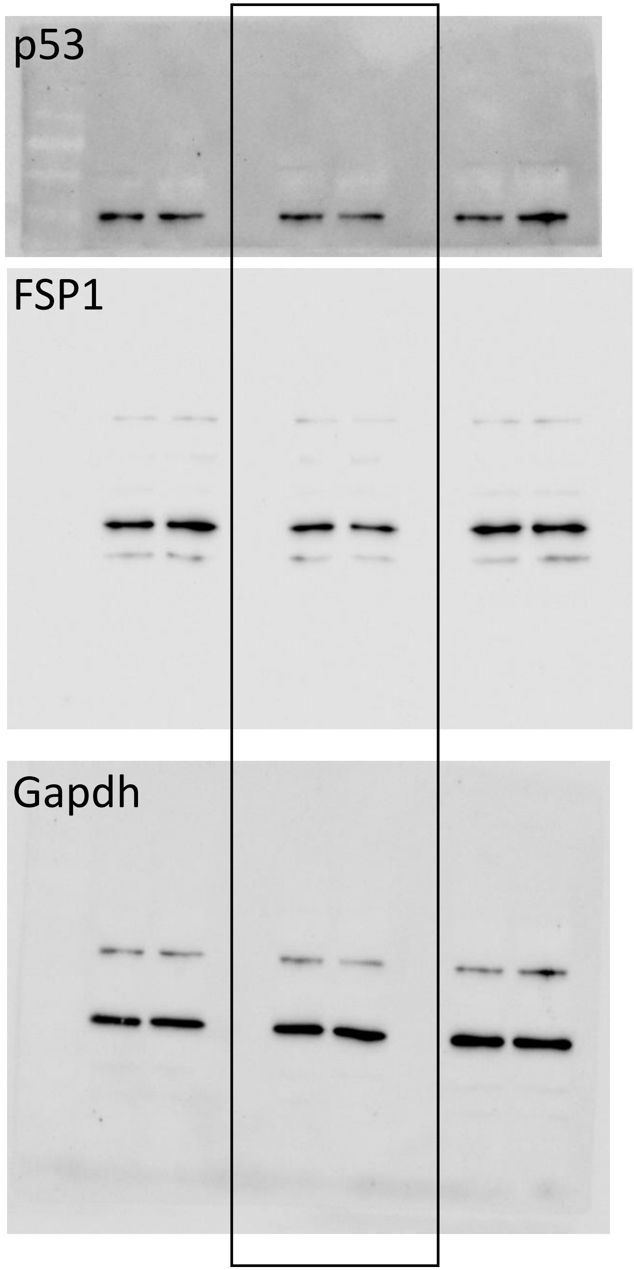

Figure 6G

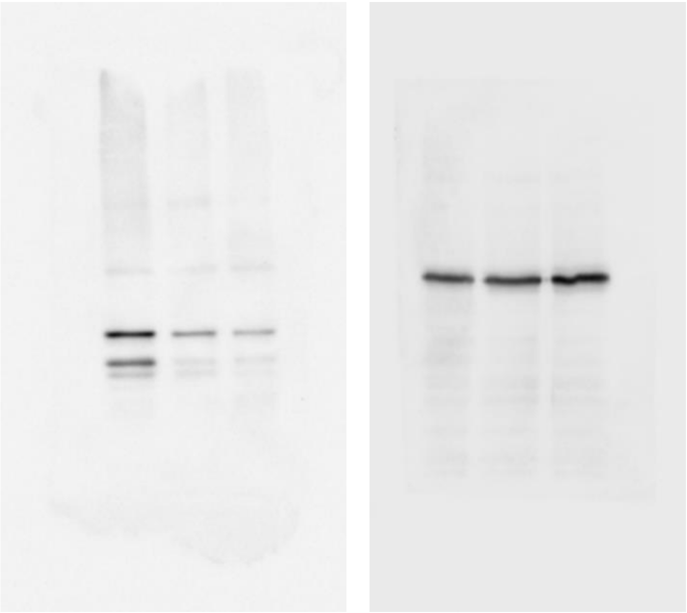

FSP1 Tubulin

Supplementary S4

E2F1

FSP1

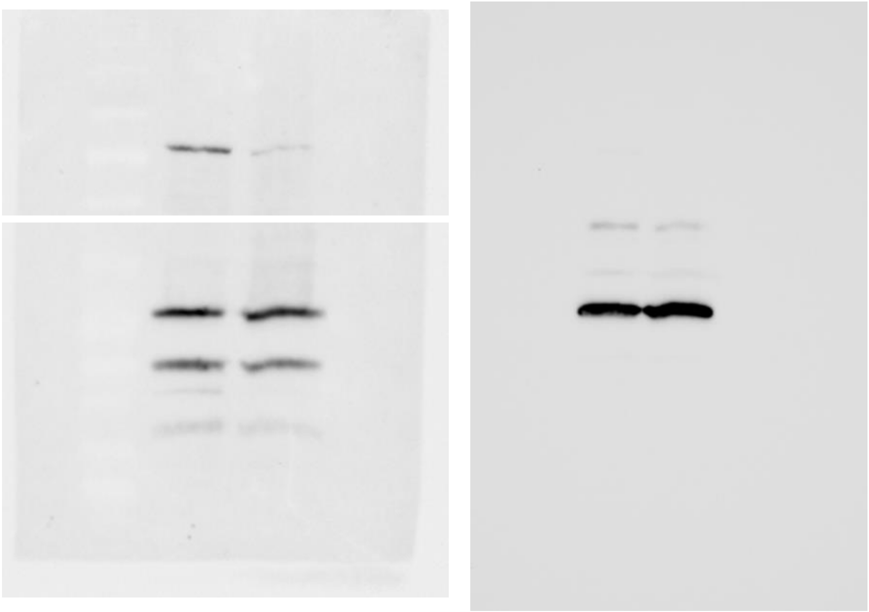

Gapdh

Supplementary S9
