# Supplementary material for: FSP1 is a predictive biomarker of osteosarcoma cells’ susceptibility to ferroptotic cell death and a potential therapeutic target
Source: Cell Death Discov. 2024 Feb 17;10:87. doi: 10.1038/s41420-024-01854-2 (PMC10874395; doi:10.1038/s41420-024-01854-2)
Supplement: Supplementary file 4 — Supplementary Information [file 41420_2024_1854_MOESM4_ESM.pdf]

Supplementary Information

MOV1-6. Wound healing assay. U2OS (MOV1 & MOV2), MG63 (MOV3 & MOV4), or HOS (MOV5 & MOV6) cells were plated in 6-well plates and were allowed to grow until 100% confluence. The cell layer was then gently scratched through the central axis using a sterile plastic tip. The width of the healing monolayer wound was recorded over 24h in a time lapse experiment (Δt = 30 min) using a THUNDER 3D Cell Imager system (Leica). Animations were generated by using the LASX software (Leica).

MOV7-10. U2OS viability in microfibers. U2OS were grown in alginate-based 3D microfibers, treated as indicated, and stained with Calcein-AM/PI, as described in the main text. Then, image acquisition was performed by using a Leica TCS SP8 Confocal microscope (Objective HC PL FLUOTAR 10x/0.30 DRY) in the xyz mode as follows:

**MOV7: CTRL**					
Dimension	*Logical size*	*Physical size*	*Start position*	*End position*	*Pixel size/Voxel size*
X	1024	1162.5 μm	0 μm	1162.5 μm	1.136 μm
Y	1024	1162.5 μm	0 μm	1162.5 μm	1.136 μm
Z	162	689.18 μm	5373.18 μm	4684 μm	4281 μm
**MOV8: iFSP1**					
Dimension	*Logical size*	*Physical size*	*Start position*	*End position*	*Pixel size/Voxel size*
X	1024	1162.5 μm	0 μm	1162.5 μm	1.136 μm
Y	1024	1162.5 μm	0 μm	1162.5 μm	1.136 μm
Z	127	539.36 μm	4991.12 μm	4451.76μm	4281 μm
**MOV9: RSL3**					
Dimension	*Logical size*	*Physical size*	*Start position*	*End position*	*Pixel size/Voxel size*
X	1024	1162.5 μm	0 μm	1162.5 μm	1.136 μm
Y	1024	1162.5 μm	0 μm	1162.5 μm	1.136 μm
Z	164	697.74 μm	5127.26 μm	4429.52μm	4281 μm
**MOV10: RSL3+iFSP1**					
Dimension	*Logical size*	*Physical size*	*Start position*	*End position*	*Pixel size/Voxel size*
X	1024	1162.5 μm	0 μm	1162.5 μm	1.136 μm
Y	1024	1162.5 μm	0 μm	1162.5 μm	1.136 μm
Z	135	573.6 μm	5061.85 μm	4488.25μm	4281 μm

3D images and animations were generated by using the LASX software (Leica).
