# Supplementary material for: FSP1 is a predictive biomarker of osteosarcoma cells’ susceptibility to ferroptotic cell death and a potential therapeutic target
Source: Cell Death Discov. 2024 Feb 17;10:87. doi: 10.1038/s41420-024-01854-2 (PMC10874395; doi:10.1038/s41420-024-01854-2)
Supplement: Supplementary file 1 — Author changes [file 41420_2024_1854_MOESM1_ESM.pdf]

UNIVERSITÀ DEL PIEMONTE ORIENTALE

UNIVERSITÀ DEL PIEMONTE ORIENTALE
CENTER FOR TRANSLATIONAL RESEARCH ON AUTOIMMUNE AND ALLERGIC DISEASE
CAAD

Cell stress & death lab
Adapt or Die

Monday, February 5, 2024

Dear Editor,

I hereby confirm my consent to include Dr Jelena Petrovic, Professor Jasmina Stojkovska, and Professor Licio Collavin as authors in the latest version of our manuscript entitled: 'FSP1 is a predictive biomarker of osteosarcoma cells' susceptibility to ferroptotic cell death and a potential therapeutic target' (CDDISCOVERY-23-1118R2).

Here below please find the response from all co-authors

Sincerely,

Prof. Marco Corazzari

Associate Professor of Applied Biology
Laboratory of Applied Biology
Department of Health Sciences (DISS)
Palazzo Bellini, V. Solaroli 17 &
Cell Stress & Death Lab
Center for Translational Research on Autoimmune and Allergic Disease (CAAD)
C.so Trieste 15A
University of Piemonte Orientale
28100 Novara - IT

Cell stress & death lab
Adapt or Die

UNIVERSITÀ DEL PIEMONTE ORIENTALE

Marco Corazzari <marco.corazzari@uniupo.it>

Author list changes - CDDISCOVERY-23-1118R2

5 messaggi

Marco Corazzari <marco.corazzari@uniupo.it> 31 gennaio 2024 alle ore 13:02
A: Elzbieta Janina Panczyszyn <elzbieta.panczyszyn@uniupo.it>, Valentina Saverio <valentina.saverio@uniupo.it>, Romina Monzani <romina.monzani@uniupo.it>, Mara Gagliardi <mara.gagliardi@uniupo.it>

Dear co-authors,

I am writing this email to ask you to confirm your consent to include Dr Jelena Petrovic, Professor Jasmina Stojkovska, and Professor Licio Collavin as authors in the latest version of our manuscript entitled: 'FSP1 is a predictive biomarker of osteosarcoma cells' susceptibility to ferroptotic cell death and a potential therapeutic target' (CDDISCOVERY-23-1118R2).

Regards,
--
***Marco Corazzari**, PhD*
Associate Professor of Applied Biology
Cell Stress & Death Lab
Department of Health Sciences – Univerità del Piemonte Orientale

---

Head of the Advanced Microscopy Facility
Center for Translational Research on Autoimmune and Allergic Disease (CAAD)
C.so Trieste 15° - 28100 Novara - IT
Phone: +39 0321 660 812
skype: marcocorazzariMC

Cell stress & death lab
Adapt or Die

Elzbieta Janina Panczyszyn <elzbieta.panczyszyn@uniupo.it> 31 gennaio 2024 alle ore 13:04
A: Marco Corazzari <marco.corazzari@uniupo.it>
Cc: Valentina Saverio <valentina.saverio@uniupo.it>, Romina Monzani <romina.monzani@uniupo.it>, Mara Gagliardi <mara.gagliardi@uniupo.it>

I confirm my consent.

Elżbieta Pańczyszyn
[Testo tra virgolette nascosto]

Valentina Saverio <valentina.saverio@uniupo.it> 31 gennaio 2024 alle ore 13:06
A: Elzbieta Janina Panczyszyn <elzbieta.panczyszyn@uniupo.it>
Cc: Marco Corazzari <marco.corazzari@uniupo.it>, Romina Monzani <romina.monzani@uniupo.it>, Mara Gagliardi <mara.gagliardi@uniupo.it>

I confirm my consent.
Valentina Saverio
[Testo tra virgolette nascosto]
--
Valentina Saverio, Ph.D
Cell Stress & Death Lab
Department of Health Sciences (DISS), University of Piemonte Orientale "A. Avogadro"
Palazzo Bellini, V. Solaroli 17, 28100 Novara - IT
Center for Translational Research on Autoimmune and Allergic Disease (CAAD)
V.le Trieste, 15 A, 28100 Novara - IT

Cell stress & death lab
Adapt or Die

Romina Monzani <romina.monzani@uniupo.it> 31 gennaio 2024 alle ore 13:11
A: Marco Corazzari <marco.corazzari@uniupo.it>

I confirm my consent.
Romina Monzani
[Testo tra virgolette nascosto]

Mara Gagliardi <mara.gagliardi@uniupo.it> 31 gennaio 2024 alle ore 13:12
A: Marco Corazzari <marco.corazzari@uniupo.it>
Cc: Elzbieta Janina Panczyszyn <elzbieta.panczyszyn@uniupo.it>, Valentina Saverio <valentina.saverio@uniupo.it>, Romina Monzani <romina.monzani@uniupo.it>

I confirm my consent.
Mara Gagliardi
[Testo tra virgolette nascosto]

UNIVERSITÀ DEL PIEMONTE ORIENTALE

Marco Corazzari <marco.corazzari@uniupo.it>

Author list changes

4 messaggi

Marco Corazzari <marco.corazzari@uniupo.it> 5 febbraio 2024 alle ore 08:53
A: Licio Collavin <lcollavin@units.it>, Jasmina Stojkovska <jstojkovska@tmf.bg.ac.rs>, Jelena Petrovic <jelenapetrovic.co@gmail.com>

Dear colleagues,

I am writing this email to ask you to confirm your formal consent to be included as co-authors in the latest version of our manuscript entitled: 'FSP1 is a predictive biomarker of osteosarcoma cells' susceptibility to ferroptotic cell death and a potential therapeutic target' (CDDISCOVERY-23-1118R2).

[Please, reply asap to this email with your response]

Regards,
--
***Marco Corazzari**, PhD*
Associate Professor of Applied Biology
Cell Stress & Death Lab
Department of Health Sciences – Univerità del Piemonte Orientale

---

Head of the Advanced Microscopy Facility
Center for Translational Research on Autoimmune and Allergic Disease (CAAD)
C.so Trieste 15° - 28100 Novara - IT
Phone: +39 0321 660 812
skype: marcocorazzariMC

Cell stress & death lab
Adapt or Die

COLLAVIN LICIO <lcollavin@units.it> 5 febbraio 2024 alle ore 09:07
A: Marco Corazzari <marco.corazzari@uniupo.it>

Dear Marco,

I consent to be included as co-author in the latest version of the manuscript entitled 'FSP1 is a predictive biomarker of osteosarcoma cells' susceptibility to ferroptotic cell death and a potential therapeutic target' (CDDISCOVERY-23-1118R2).

Best regards,
Licio

--
Prof. Licio Collavin, PhD
Dip. Scienze della Vita | Dept. of Life Sciences
Università degli Studi di Trieste | University of Trieste
[Testo tra virgolette nascosto]

Jelena Petrovic <jelenapetrovic.co@gmail.com> 5 febbraio 2024 alle ore 09:22
A: Marco Corazzari <marco.corazzari@uniupo.it>

Dear Marco,

I consent.

Best regards,

Jelena
[Testo tra virgolette nascosto]

Jasmina Stojkovska <jstojkovska@tmf.bg.ac.rs> 5 febbraio 2024 alle ore 11:46
A: Marco Corazzari <marco.corazzari@uniupo.it>

Dear Marco,

I hereby confirm my consent to be included as a co-author in the latest
version of our manuscript.

All the best,
Jasmina

On Mon, February 5, 2024 8:53 am, Marco Corazzari wrote:
> Dear colleagues,
>
>
> I am writing this email to ask you to confirm your formal consent to be
> included as co-authors in the latest version of our manuscript entitled:
> 'FSP1 is a predictive biomarker of osteosarcoma cells' susceptibility to
> ferroptotic cell death and a potential therapeutic target'
> (CDDISCOVERY-23-1118R2).
>
>
> *[Please, reply asap to this email with your response]*
>
>
> Regards,
> --
> *Marco Corazzari, PhD*
>
>
> *Associate Professor of Applied BiologyCell Stress & Death LabDepartment
> of Health Sciences – Univerità del Piemonte
> Orientale____Hea
> d of the Advanced Microscopy FacilityCenter for Translational Research on
> Autoimmune and Allergic Disease (CAAD)C.so Trieste 15° - 28100 Novara -
> ITPhone: +39 0321 660 812skype: marcocorazzariMC*
>
>

Dr. Jasmina Stojkovska, Senior Research Associate
Faculty of Technology and Metallurgy
University of Belgrade
Belgrade, Serbia
phone +381(0)11 3303-609
fax +381(0)11 3370-387
